# Tubulin evolution in insects: gene duplication and subfunctionalization provide specialized isoforms in a functionally constrained gene family

**DOI:** 10.1186/1471-2148-10-113

**Published:** 2010-04-27

**Authors:** Mark G Nielsen, Sudhindra R Gadagkar, Lisa Gutzwiller

**Affiliations:** 1 Department of Biology University of Dayton, Dayton, OH 45467, USA; 2 Department of Natural Sciences Central State University 1400 Brush Row Road Wilberforce OH 45384, USA; 3 Division of Developmental Biology University of Cincinnati Children's Hospital 3333 Burnet Ave. Cincinnati, OH 45229, USA

## Abstract

**Background:**

The completion of 19 insect genome sequencing projects spanning six insect orders provides the opportunity to investigate the evolution of important gene families, here tubulins. Tubulins are a family of eukaryotic structural genes that form microtubules, fundamental components of the cytoskeleton that mediate cell division, shape, motility, and intracellular trafficking. Previous *in vivo *studies in *Drosophila *find a stringent relationship between tubulin structure and function; small, biochemically similar changes in the major alpha 1 or testis-specific beta 2 tubulin protein render each unable to generate a motile spermtail axoneme. This has evolutionary implications, not a single non-synonymous substitution is found in *beta 2 *among 17 species of *Drosophila *and *Hirtodrosophila *flies spanning 60 Myr of evolution. This raises an important question, How do tubulins evolve while maintaining their function? To answer, we use molecular evolutionary analyses to characterize the evolution of insect tubulins.

**Results:**

Sixty-six alpha tubulins and eighty-six beta tubulin gene copies were retrieved and subjected to molecular evolutionary analyses. Four ancient clades of alpha and beta tubulins are found in insects, a major isoform clade (*alpha 1, beta 1*) and three minor, tissue-specific clades (*alpha 2-4, beta 2-4*). Based on a *Homarus americanus *(lobster) outgroup, these were generated through gene duplication events on major beta and alpha tubulin ancestors, followed by subfunctionalization in expression domain. Strong purifying selection acts on all tubulins, yet maximum pairwise amino acid distances between tubulin paralogs are large (0.464 substitutions/site beta tubulins, 0.707 alpha tubulins). Conversely orthologs, with the exception of reproductive tissue isoforms, show little sequence variation except in the last 15 carboxy terminus tail (CTT) residues, which serve as sites for post-translational modifications (PTMs) and interactions with microtubule-associated proteins. CTT residues overwhelming comprise the co-evolving residues between *Drosophila *alpha 2 and beta 3 tubulin proteins, indicating CTT specializations can be mediated at the level of the tubulin dimer. Gene duplications post-dating separation of the insect orders are unevenly distributed, most often appearing in major *alpha 1 *and minor *beta 2 *clades. More than 40 introns are found in tubulins. Their distribution among tubulins reveals that insertion and deletion events are common, surprising given their potential for disrupting tubulin coding sequence. Compensatory evolution is found in *Drosophila beta 2 *tubulin cis-regulation, and reveals selective pressures acting to maintain testis expression without the use of previously identified testis cis-regulatory elements.

**Conclusion:**

Tubulins have stringent structure/function relationships, indicated by strong purifying selection, the loss of many gene duplication products, alpha-beta co-evolution in the tubulin dimer, and compensatory evolution in *beta 2 *tubulin cis-regulation. They evolve through gene duplication, subfunctionalization in expression domain and divergence of duplication products, largely in CTT residues that mediate interactions with other proteins. This has resulted in the tissue-specific minor insect isoforms, and in particular the highly diverse *α3*, *α4*, and *β2 *reproductive tissue-specific tubulin isoforms, illustrating that even a highly conserved protein family can participate in the adaptive process and respond to sexual selection.

## Background

Proteins vary in the stringency of their structure/function relationships, which may affect their ability to participate in the adaptive process [1]. Nature has ready opportunity to shape the phenotype through selection on proteins that show non-synonymous allelic variation, for example esterases [2] and glycolytic enzymes [3]. Other proteins, for example actins, show little amino acid variation (~5-7% across metazoans, [4]), and tend to loose function entirely rather than provide altered function in response to change in their amino acid sequence [5]. Such proteins may not typically admit allelic variation, which raises an old, but important question: is selection a shaper of diversity, or merely an executioner [6]?

One of the best-studied proteins with respect to its structure/function relationship is tubulin. *In vivo *studies of alpha and beta tubulin in the *Drosophila melanogaster *spermtail axoneme find that small changes in the amino acid sequence of the major alpha 1 or testis-specific beta 2 tubulin protein render each unable to generate a motile axoneme [7-10]. This stringency has evolutionary implications; comparisons of *beta 2 *sequences among different species of *Drosophila *find not a single non-synonymous substitution, indicating the protein has not changed in sequence for more than 60 million years [11]. Together these results indicate that only rarely does *beta 2 *participate in the adaptive process.

For proteins with stringent structure/function relationships, evolving while maintaining function is problematic. Gene duplication is a fundamental mechanism in answer to this problem [12], yet without additional changes, in expression domain and/or in the proteins with which it co-functions, a duplicate copy will have the same function as the original, will experience the same selective regime as the original and so will not evolve.

Here we characterize insect tubulin evolution, to identify events that release tubulins to evolve, and to more generally serve as a model for the evolution of functionally constrained proteins. Tubulins are a family of eukaryotic structural proteins that comprise microtubules, fundamental components of the spindle in cell division, the axoneme in cilia and flagella, mediators of cell shape, and dynein/kinesin-based cell trafficking [13-15]. Two members of the tubulin family, alpha and beta tubulin, form a dimer that is the building block of the microtubule. All eukaryotes contain at least one major alpha (*α1*) and beta (*β1*) tubulin. In addition, *Drosophila melanogaster *express minor, tissue-specific isoforms in the motile spermtail axoneme (*β2*), pre-adult tissues (*β3, β4*, and *α2*), and the ovary (*α4*) [16,17].

We studied tubulin evolution in two hemimetabolous insect orders, Phthiraptera (*Pediculus humanus corporis*, body louse) and Hemiptera (*Acyrthosiphon pisum*, pea aphid), and four holometabolous orders, Hymenoptera (*Apis millifera *honeybee, *Nasonia vitripennis *jewel wasp), Coleoptera (*Tribolium castenatum *flour beetle), Lepidoptera (*Bombyx mori *silkmoth), and Diptera (*Aedes aegypti, Anopheles gambiae*, and *Drosophila melanogaster, D. sechellia, D. yakuba, D. erecta, D. simulans, D. mojavensis, D. grimshawi, D. ananassae, D. persimilis, D. psuedoobscura, D. virilis, D. willistoni*). These orders represent well over 80% of the diversity in all insect species [18]. Their evolutionary relationships are not controversial, and each of these orders is considered to be monophyletic [19]. They are known to be quite ancient, the origin of these insect orders has been dated to be >300 Mya using a molecular clock [20], with the oldest beetle (Coleopteran) fossils from the Lower Permian (about 265 million years ago [21]) and the earliest fly (Diptera) fossil from the Upper Triassic of the Mesozoic geological period, some 225 million years ago [22].

We find four clades of alpha and beta tubulins in insects that, for the most part, do not evolve without a gene duplication event. Yet gene duplication is not sufficient to release tubulin evolution, most duplication products are lost, and major tubulin duplication products do not evolve unless followed by subfunctionalization in expression domain. Subfunctionalization has resulted in a number of reproductive tissue-specific tubulins that are diverse in sequence, particularly in CTT residues that mediate integrations with other proteins. Together these results indicate that tubulin evolution is constrained, yet tubulins can in fact participate in the adaptive process.

## Methods

### Sequence retrieval

Insect tubulins were obtained through BLAST [23] searches of the sequenced insect genome databases http://flybase.bio.indiana.edu/ and NCBI http://www.ncbi.nlm.nih.gov/ databases using *Drosophila melanogaster *tubulin cDNAs as query sequences. Tubulin exon/intron structure was determined by aligning retrieved genomic sequences to their *Drosophila *cDNA orthologs using Sequencher 4.1 (Gene Codes Corporation [24]).

### Genealogical reconstruction

DNA sequence alignments were made using ClustalW [25] in the MEGA v. 4.0 software package [26]. Translated sequences were aligned using BLOSUM [27] (gap opening penalty 100, extension penalty 0.1), refined by hand, and untranslated for genealogical analysis using the Bayesian method as implemented in Mr. Bayes v. 3.1.2 [28]). For both alpha and beta tubulins a GTR model was used with 4 rate categories, gamma corrections were estimated by the program, and gaps were coded. For beta tubulins, the analysis was done using first and second codon positions (88 sequences, 947 sites, gamma correction α = 0.542); for alpha tubulins zero-fold degenerate codon positions (69 sequences, 811 sites, gamma correction α = 0.486). Analyses were run until the standard deviation of split frequencies was below 0.01, for the alpha tubulins 4,000,000 generations and beta tubulins 1,500,000 generations. A 25% burnin was performed [28], and the majority-rule consensus tree is reported.

### Pairwise amino acid distances

The average and maximum pairwise amino acid distances (number of amino acid differences/site) between paralogs and orthologs are presented, these were generated using the Poisson correction method in MEGA v. 4 [26]; standard error estimates were obtained by a bootstrap procedure (2000 replications).

### Test of selection

Since tubulins do not appear to be evolving at appreciable rates, they are probably under severe purifying selection. On the other hand, the carboxy terminus tails, which mediate tubulin functional specializations via interactions with other proteins, and are released from protein folding constraints because they lie on the surface of the MT [29], are the most rapidly evolving, and possibly under positive selection. These two hypotheses were tested, first by means of estimating dN (the number of nonsynonymous substitutions per nonsynonymous site), dS (the number of synonymous substitutions per synonymous site), and the ratio dN/dS (ω) by the ML method as implemented in PAML v4.3b http://abacus.gene.ucl.ac.uk/software/paml.html[30], and then by means of a likelihood ratio test comparing the null model H_0 _with ω = 1 fixed and the alternative model H_1 _with ω estimated from the data.

### Rate tests

The tubulins were tested for the molecular clock, to determine: 1) if paralogous tubulins resulting from ancient duplication events (preceding separation of insect orders) evolve at the same rate, 2) if orthologous tubulins evolve at the same rate, and 3) if paralogous tubulins resulting from recent duplication events (post-dating separation of the insect orders) diverge following duplication. All rate tests were performed using Tajima's relative rates test in MEGA v. 3.0 [26,31].

### Test of co-evolution

Stringency in tubulin structure/function relationships may result from the need to maintain proper lateral and longitudinal contacts between alpha and beta tubulin in the microtubule, such that the alpha and beta tubulins must co-evolve for either tubulin component to evolve. Sato's mirror-tree method [32] compares partial correlation coefficients between candidate co-evolving proteins' distance matrices to identify co-evolution. This test requires 1) multiple sequences for comparison, 2) sequence variation among those sequences, and 3) knowledge that the alpha and beta isoforms are co-expressed in a cell. These conditions are met only by the *Drosophila α2 *and *β3 *tubulins, which co-function in visceral mesoderm, the testis cyst cells, and pre-adult sensory neurons [7,8].

### Evolution of *Drosophila Beta 2 *tubulin cis-regulation

An opportunity to study cis-regulatory aspects of tubulin evolution is provided by the *D. melanogaster β2 *gene. Three aspects of *Dmβ2 *regulation have been identified, the β2UE1 element, required for testis-specific gene expression, and the β2UE2 and β2DE1 elements for proper expression levels [33]; these elements are identifiable in most *Drosophila *species. The core promoter does not contain a TATA box, but uses an Inr sequence found in many TATA-less promoters. We identified these sequences in *Drosophila *species by scanning the 1000 base pairs 5' to transcription start for identical and degenerate sequence matches to *Dmβ2 *regulation, using Sequencher.

To determine if *Drosophila β2 *is expressed in the testis in species in which these sequences were not found, a PCR approach was used. RNA was extracted from 20 pairs of dissected testes using a guanidine hydrochloride, phenol/chloroform method [34], and DNAase treated to remove DNA contaminants (New England Biolabs). *Beta 2*-specific primers were used in a reverse transcription reaction, followed by PCR amplification, to identify *β2 *mRNA in testes. For a negative control, PCR was performed on the same RNA template, minus the reverse transcription reaction.

## Results

### Sequence retrieval and genealogical reconstruction

A total of 86 beta tubulins and 66 alpha tubulins were obtained through blast searches of the completed insect genome projects and NCBI databases (Tables 1, 2; Additional File 1). *Homarus americana *(American lobster; Crustacea, Decapoda) tubulins were used as an outgroup in the genealogical reconstruction, because *Homarus *is the closest relation to insects for which the full complement of tubulins has been identified. *Homarus *has two major beta tubulin isoforms and three major alpha tubulins. Based on this outgroup, duplication products of major alpha and beta tubulin isoforms gave rise to the insect major and minor tubulins.

**Table 1:** Insect beta tubulin sequence features.

Isoform	Function	Average and maximum pairwise distances			-COOH terminus sequence			Post-translational modification
								sites/Conserved
		*Drosophila*	Mosquito	All Insects				sequence features
***β1***	**Major ** **isoform**	**0.000 ** **+/- ** **0.000** **(n = 12)**	**0.002 ** **+/- ** **0.002** **(n = 2)**	**Average** **0.011 ** **+/- ** **0.003** **(n = 9)** **Maximum ** **0.027** **(n = 10)**	***Ha*β1a EATADDEAE FEEEGEVEGE YA** ***Ha*β1b EATADDEAE FEEEGEVEGE YD** ***Dm*β1 EATADEDAE FEEEQEAEVD EN** ***Ae*β1 EATADEDAE FDEEQEAEVD EN** ***Ag*β1 EATADEDAE FDEEQEAEVD EN** ***Bm*β1a EATADEDAE FDEEQEQEIE DN** ***Bm*β1b EATADEDAE FDEEQEQEIE EH** ***Tc*β1 EATADEDAE FDEEQEAEVD EN** ***Nv*β1a -** ***Nv*β1b TMNGPRDAP DEDVEVVEEE LRD** ***Am*β1 EATADEDAE FDEEQEAEVD EN** ***Ap*β1 EATADEEAE FDEEQEQEVD EN** ***Ph*β1 EATADEDAE FDEEQEEVVD EN**			**PTM sites** Polyglutamylation - yes Polyglycylation - yes Phosphorylation - yes

***β2***	**Testis-** **specific ** **isoform**	**0.000 ** **+/- ** **0.000** **(n = 12)**	**0.060 ** **+/- ** **0.011** **(n = 2)**	**Average** **0.085 ** **+/- ** **0.009** **(n = 9)** **Maximum ** **0.464** **(n = 17)**	***Dm*β2 EATADEEGE FDEDEEGGGD E** ***Ae*β2 EATADEEGE FDEEEEGGEE** ***Ag*β2 EATADDEGE MDEEEEGGED** ***Bm*β2 DATADDEGE FDEEAEEGLE E** **Tcβ2a DATAEEEGE FDEEEEGDNE GEN** ***Tc*β2b DAEVDEEYG DEDETEEDKF EEET** ***Nv*β2a EATAEEDTE FDEDEGENEG N** ***Nv*β2b EATADEFAD YEEDEEEEED YA** ***Nv*β2c EATTEE--D FETEDAGDD FETCDQE** ***Am*β2a EATAEEEGE FDEEEEGEGE HP** ***Am*β2b EATAEDEGE FDEEEETEK** **Apβ2a DATVDEDGE GDDDEEDADA** **Apβ2b EATIDETGE-EDEDEDADA** **Apβ2c DATVDEEGE GDDDDEDAEA** **Apβ2d EATVDAPGG VNEE** ***Ph*β2a EATADEEGE DEEDEGGED** ***Ph*β2b EATSYEYDE DEGEENEVEE EEEKEMTNWL PA**			**PTM sites** Polyglutamylation - yes, except *Ap*β2d Polyglycylation -- yes, except *Ap*β2d Phosphorylation -- yes **Conserved sequence features** Axoneme motif - yes, except *Ag*β2, *Tc*β2b, *Nv*β2a-c, *Ap*β2a-d, *Ph*β2a, b Gly^56^-- yes, except *Ph*β2b

***β3***	**Minor ** **isoform ** **expressed in ** **variety of ** **pre-adult ** **mesodermal ** **and neural ** **domains**	**0.009 ** **+/- ** **0.002** **(n = 10)**	**0.014 ** **+/- ** **0.005** **(n = 2)**	**Average ** **0.092 ** **+/- ** **0.010** **(n = 9)** **Maximum ** **0.148** **(n = 9)**	***Dm*β3 EATADDEFD PEVNQEEVEG DCI** ***Ae*β3 EATADDEFE QEECADEMEG ECV** ***Ag*β3 EATADDEFE QEDCQDEMEG ECV** ***Bm*β3 EATAEDDTE FDQEDLEELA QDEHHD** ***Tc*β3 EATADEEYE AEEEAAADDF NC** ***Nv*β3 EATTEEDFE TEDAGDDFET CDQE** ***Am*β3 EATAEEDFE AEECADDFET CDQE** ***Ap*β3 EASVDEEYI EEEETEETDM CD** ***Ph*β3 LYISTIIKI**			**PTM sites** Polyglutamylation -- yes Polyglycylation -- yes, except *Tc*β3, *Nv*β3, *Am*β3, *Nv*β3, *Ap*β3 Phosphorylation - yes **Conserved sequence features** Nucleotide-binding domain amino acid insert (aa^56^) - yes

***β4***	**Minor ** **isoform, ** **pre-adult ** **tissues in ** ***Dm*, absent ** **in *Bm)***	**0.032 ** **+/- ** **0.005** **(n = 11)**	**0.104 ** **+/- ** **0.012** **(n = 3)**	**Average ** **0.136 ** **+/- ** **0.015** **(n = 5)** **Maximum ** **0.186** **(n = 6)**	***Dm*β4 EATADDEVE FDDEQAEQEG YESEVLQNGN GE** ***Ae*β4a EASADDYVE GEHDFDDEEE IQQ** ***Ae*β4b DASVEDYED GEEMIEEEGE QHVE** ***Ag*β4 DAEVEDYDE MEEIPEEEQQ QQQE** ***Ap*β4 EATAEEVEF DDEEVVEEVD DNKDY** ***Ph*β4 VRSSLHLSN AANIEIQKNE ILNRNT**			**PTM sites** Polyglutamylation - ? Polyglycylation - ? Phosphorylation - yes

Features of the four beta tubulin isoforms identified in insects are presented. The function and/or expression domain of each sequence in *D. melanogaster *[16,17] and *B. mori *[35], the two insects in which tubulin expression and function have been studied, are presented in Column 2. Average and maximum pairwise distance calculations in Column 3 refer to the average # amino acid differences/site among conserved isoforms, and the maximum pairwise distance between any two orthologs, including divergent duplication products, respectively. For "all insects", the only *Drosophila *species included is *Dm*, to avoid a Dipteran skew in the results. CTT sequences are presented in Column 4, for purposes of inspection as they constitute ~50% of the differences among tubulins. Tubulin post-translational modifications (PTMs) occur on sequence motifs whose presence and absence are presented in the Column 5. Polyglutamylation and polyglycylation sequence motifs are degenerate, the "?" indicates that a potentially modifiable, but experimentally uncharacterized residue(s) is present for these PTMs. Unusual sequence features or motifs known to mediate specific tubulin functional specializations are also noted. The full-length sequences for *Nvβ1, Phβ3, Dpβ3, Dsβ3 *were not available. Key: *Pediculus humanus corporisPh, Acyrthosiphon pisum Ap, Apis millifera Am*, *Nasonia vitripennis Nv*, *Tribolium castenatum Tc, Bombyx mori Bm*, *Aedes aegypti Ae*, *Anopheles gambiae Ag*, *Drosophila melanogaster Dm, D. sechellia Dc, D. yakuba Dy, D. erecta De, D. simulans Ds, D. mojavensis Do, D. grimshawi Dg, D. ananassae Da, D. persimilis Dp, D. psuedoobscura Du, D. virilis Dv, D. willistoni Dw*).

**Table 2:** Insect alpha tubulin sequence features.

Isoform	Function	Average and maximum pairwise distances			-COOH terminus sequence			Post-translational modification sites/Conserved sequence features
				
		*Drosophila*	Mosquito	All Insects				
***α1***	**Major ** **isoform** **α1a: ** **Somatic ** **and Testis ** **function ** **in *Dm*, ** **somatic ** **only in *Bm*** **α1b: low ** **level somatic ** **in *Dm***	**0.000 ** **+/- ** **0.000** **(n = 17)**	**0.004 ** **+/- ** **0.002** **(n = 4)**	**Average** **0.010 ** **+/- ** **0.003 ** **(n = 9)** **Maximum ** **0.086** **(n = 18)**	***Ha*α1a DLAALEKDY EEVGVDSADA EGEEEGEEY** *** Ha*α1b DLAALEKDY EEVGMDSADG EDIEGGDEY** *** Ha*α1c DLATLEKDY EEVGIDTADG EDDEEANDY** *** Dm*α1a DLAALEKDY EEVGMDSGDG EGEGAEEY** *** Dm*α1b DLAALEKDY EEVGMDSGDG EGEGAEEY** *** Ae*α1a DLAALEKDY EEVGMDSGEG EGEGAEEY ** ***Ae*α1b DLAALEKDY EEVGMDSGEG EGEGAEEY** *** Ag*α1a DLAALEKDY EEVGMDSGEG EGEGAEEY** *** Ag*α1b DLAALEKDY EEVGMDSGDG EGEGAEEY** *** Bm*α1 DLAALEKDY EEVGMDSAEG EGEGAEEY** ***Tc*c1 DLAALEKDY EEVGMDSGEG EGEGGEEY** ***Am*α1a DLAALEKDY EEVGMDSAEG EGEGAEEY** ***Am*α1b DLAALEKDY EEVGMDSAEG EGEGAEEY** ***Nv*α1a DLAALEKDY EEVGMDSVEG EGEGAEEY** ***Nv*α1b DLAALEKDY EEVGMDSTEG EGEGAEEY** ***Nv*α1c DLAALEKDY EEVGMDSTEG EGEGAEEY** ***Ap*α1a DLAALEKDY EEVGMDSVEG EGEGGEEY** ***Ap*α1b DLAALEKDY EEVGLDSVEG QFDEGVEDF** ***Ap*α1c DLAALEKDY EEVGMDSTEG DGEAGEEEI** ***Ph*α1a DLAALEKDY EEVGMDSVEG EGEGGDEY**			**PTM sites** Polyglutamylation -- yes, except *Ap*α1a Polyglycylation - yes, except *Ap*α1a Acetylation -- yes, except *Tc*α1 Detyrosination -- yes, except *Ap*α1a, *Ap*α1c, *Ph*α1a Phosphorylation - no Palmitoylation - yes

***α2***	**Minor ** **isoform often co-** **expressed with β3 in ** **variety of ** **pre-adult ** **mesodermal ** **and neural ** **domains in ** ***Dm *and *Bm***	**0.014 ** **+/- ** **0.004** **(n = 10)** **Absent ** **in *Dp *** **and *Du***	-	**Average** **0.069 ** **+/- ** **0.008** **(n = 6)** **Maximum ** **0.170** **(n = 6)**	***Dm*α2 DLAALEKDY EEVGIDSTTE LGEDEEY** ***Ae*α2 DLAALEKDY EEVGVDSTEE VGEGDEY** ***Bm*α2 DLAALEKDY EEVGVDSTEG ELDEENEY** ***Tc*α2 DLAALEKDY EEVAVDSIEG EGDEGDEY** ***Am*α2 DLAALELDY REVQEDATNT DDEEEY** ***Ph*α2 DLAALEKDY EEVGIDSVEE VGEGDEY**			**PTM sites **Polyglutamylation - ? Polyglycylation - ? Acetylation - yes Detyrosination - yes Phosphorylation - no Palmitoylation - yes

***α3***	**Testis-** **specific ** **isoform in ** ***Bm*, absent ** **in *Dm***	**-**	**-**	**Average ** **0.231 ** **+/- ** **0.003** **(n = 2)** **Maximum ** **0.231** **(n = 2)**	***Bm*α3 DLAALERDY DEVAIETSDM QPGADDEL** ***Tc*α3 DLAMLEKDY EEVSIDDIE**			**PTM sites** Polyglutamylation - ? Polyglycylation - ? Acetylation - no Detyrosination - no Phosphorylation - no Palmitoylation -- yes *Tc*α3, no *Bm*α3

***α4***	**Ovary-** **specific ** **isoform in ** ***Dm, *absent ** **in *Bm***	**0.087 ** **+/- ** **0.009** **(n = 12)**	**0.650 ** **+/- ** **0.043** **(n = 2)**	**Average ** **0.560 ** **+/- ** **0.028** **(n = 4)** **Maximum ** **0.707** **(n = 5)**	***Dm*α4 NIAVLERDF EEVGLDN*AE*E GGDEDFDEF** ***Ag*α4a DLACLERDY EEVAGDTVAS GEEYYDDDEY** ***Ag*α4b NIRTLIKDY EEI** ***Tc*α4 DLTALVLDY KEVDSD** ***Am*α4 DMMTLINDY KEIEK** ***Ph*α4 DLAALEKDY EEVGMDSVEG EGEGGEEM**			**PTM sites** Polyglutamylation -? Polyglycylation -? Acetylation - no Detyrosination -- yes, except *Ag*α4a, *Ag*α4b, *Dy*α4, *Dv*α4, *Ds*α4, *Do*α4, *Dm*α4, *Dg*α4, *De*α4, *Da*α4, *Tc*α4, *Am *c4, *Ph *c4 Phosphorylation -- yes except *Tc*α4 Palmitoylation -- yes except *Tc*α4, *Am*α4

Features of the four alpha tubulin isoforms identified in insects are presented. The function and/or expression domain of each sequence in *D. melanogaster *[16,17] and *B. mori *[35], the two insects in which tubulin expression and function have been studied, are presented in Column 2. Average and maximum pairwise ("All Insects" only) distance calculations in Column 3 refer to the average # amino acid differences/site among conserved isoforms, and the maximum pairwise distance between any two orthologs, including divergent duplication products, respectively. For "all insects", the only *Drosophila *species included is *Dm*, to avoid a Dipteran skew in the results. CTT sequences are presented in Column 4, for purposes of inspection as they constitute ~50% of the differences among tubulins. Tubulin post-translational modifications (PTMs) occur on sequence motifs whose presence and absence are presented in the Column 5. Polyglutamylation and polyglycylation sequence motifs are degenerate, the "?" indicates that a potentially modifiable, but experimentally uncharacterized residue(s) is present for these PTMs. Unusual sequence features or motifs known to mediate specific tubulin functional specializations are also noted. The full-length sequences for *Nvβ1, Phβ3, Dpβ3, Dsβ3 *were not available. Key: *Pediculus humanus corporisPh, Acyrthosiphon pisum Ap, Apis millifera Am*, *Nasonia vitripennis Nv*, *Tribolium castenatum Tc, Bombyx mori Bm*, *Aedes aegypti Ae*, *Anopheles gambiae Ag*, *Drosophila melanogaster Dm, D. sechellia Dc, D. yakuba Dy, D. erecta De, D. simulans Ds, D. mojavensis Do, D. grimshawi Dg, D. ananassae Da, D. persimilis Dp, D. psuedoobscura Du, D. virilis Dv, D. willistoni Dw*).

### Beta tubulins

**Figure 1:**
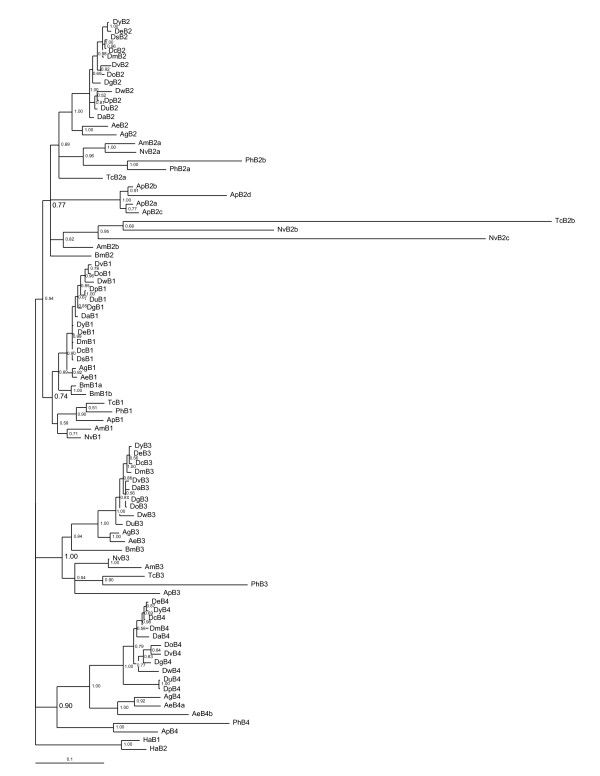
**Beta tubulin genealogy**. Bayesian reconstruction of insect beta tubulin evolutionary relationships. Eighty-six tubulins were analyzed with *Homarus americanus *(Crustacea, Decapoda) beta tubulins as the outgroup. There are four beta tubulin clades ancestral to insects, the posterior probability scores in support of these clades are in larger font in the figure: *β1 *(0.74), *β2 *(0.77), *β3 *(1.00) and *β4 *(0.90). Removal of the 5 most divergent tubulins (*Tcβ2b, Nvβ2b, c, Phβ4*, and *Apβ4*) results in support >0.97 for each clade.

**Figure 2:**
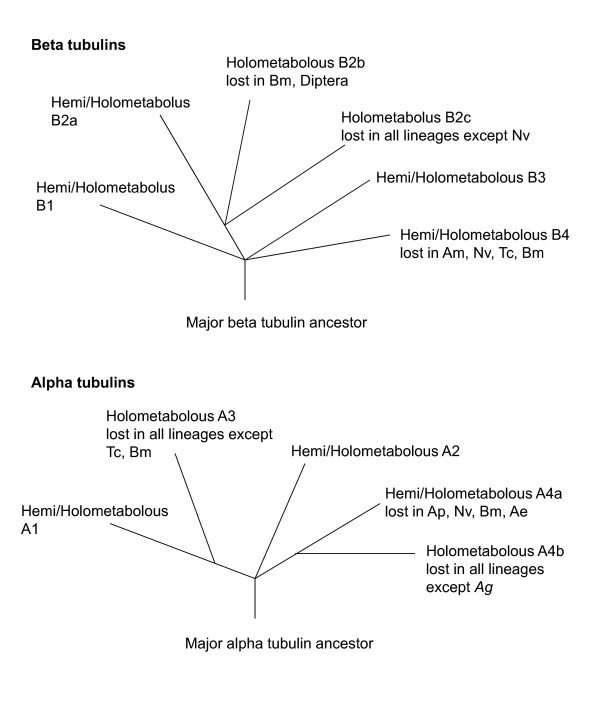
**Summary of tubulin isoform relationships**. Each of the *β1, β2, β3*, and *β4 *isoforms is represented in both hemimetabolous and holometabolous insect taxa, indicating they evolved prior to the separation of these taxa. The *β2 *isoform duplicated in holometabolous insects following their separation from hemimetabolous insects, based on the clade containing *Amβ2b, Nvβ2b, Nvβ2c, Tcβ2b*. The *β2b *isoform was lost in the Lepidoptera/Diptera ancestor, and the *β2c *isoform was lost in every holometabolous taxa except *Nv*. The *β4 *isoform is represented in hemimetabolous insects and Diptera, indicating independent losses in Hymenoptera, Coleoptera, and Lepidoptera. Each of the *α1*, *α2*, and *α4 *isoforms are represented in both hemimetabolous and holometabolous insect taxa, indicating they evolved prior to the separation of these taxa. The *α3 *isoforms, present in *Tc and Bm*, fall within the *α1 *isoforms, suggesting its origin in a duplication event in the common ancestor of Coleoptera, Lepidoptera, and Diptera that was lost in Dipterans.

There is posterior probability support (provided in parentheses for the remainder of this section) for four monophyletic beta tubulin clades in insects (Figs. 1, 2). Orthologs of the *Dmβ1 *isoform (0.74) form a clade of major somatic isoforms, the most conserved in sequence of the insect beta tubulins (Table 1). This gene duplicated in a *Bombyx *ancestor, giving rise to *Bmβ1a *and *Bmβ1b *(1.0).

A second clade consists of orthologs to the *Dmβ2 *isoform (0.77), which is testis-specific in both *Bm *and *Dm *and supports the motile axoneme [16,17,35]. Insect β2 tubulins share a Gly^62 ^which mediates doublet microtubule interactions [36], and a carboxy terminus axoneme motif "EGEFXXX" (X = Asp or Glu, [37,38]), which serves as a substrate for polyglutamylation and polyglycylation [39,40], post-translational modifications characteristic of motile axonemes. Conversely, the Thr^61 ^Gly^62 ^Ala^63 ^motif, which contributes to the extreme length of the *D. melanogaster *spermtail [10], is a unique feature of the *Drosophila *β2 protein. *Beta 2 *duplicated a number of times, in a *Pediculus *ancestor (*Phβ2a, b*: 1.0), an *Acyrthosiphon *ancestor (*Apβ2a-d*: 1.0), and in a holometabolous ancestor that is not resolved but inferred by the presence of multiple *β2 *genes in these taxa (*Amβ2b; Nvβ2b, c*; *Tcβ2b*: (0.82)) and was followed by losses of the *β2b *and *β2c *products in most taxa (Figs. 1, 2).

A third clade consists in orthologs of the *Dmβ3 *isoform (1.00), expressed in pre-adult visceral mesoderm, the testis cyst cells, and sensory neurons [16,17,35]. *Beta 3 *orthologs contain a 6 codon insertion (5 in *Ph*β3) in the internal variable region of the gene, and are the only beta tubulins that have not duplicated in insects. A fourth clade consists in orthologs of the *Dmβ4 *isoform (0.90), which is expressed in pre-adult tissues [16,17]. *Beta 4 *orthologs are the most variable beta tubulins in sequence and in representation among insects, having been lost in the *Tc, Am*, and *Bm *lineages. One *Beta 4 *duplication event is found in insects, in an *Aedes *ancestor (1.0).

### Alpha tubulins

**Figure 3:**
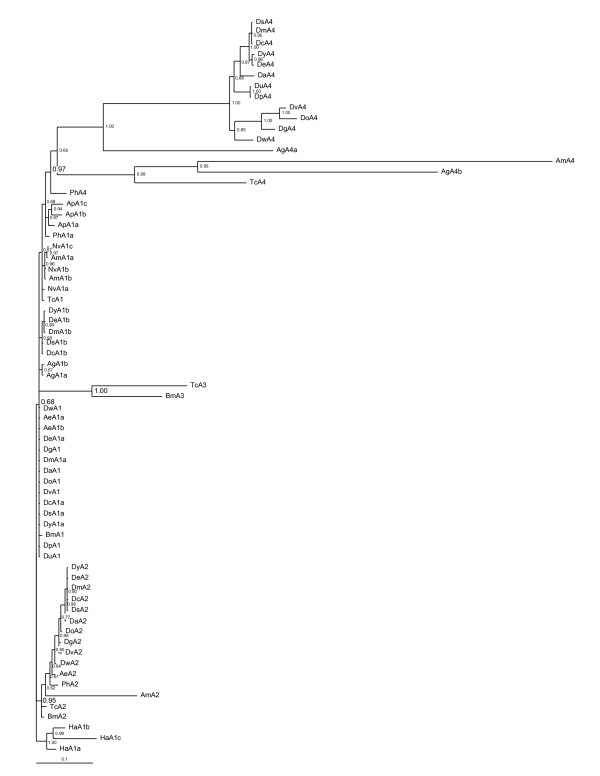
**Alpha tubulin genealogy**. Bayesian reconstruction of insect alpha tubulin evolutionary relationships. Eighty-four tubulins were analyzed with *Homarus americanus *(Crustacea, Decapoda) alpha tubulins as the outgroup. There are four alpha tubulin clades ancestral to insects, the posterior probability scores in support of these clades are in larger font in the figure: *α1 *(0.68) which contains numerous polytomies; *α2 *(0.95); *α3 *(1.0) present in *Bombyx *and *Tribolium*; and *α4 *(0.97).

There is support for four alpha tubulin insect clades, though their relationships are less resolved than the beta tubulins (Figs. 2, 3). There is a major *α1 *clade with numerous polytomies (0.68). These are orthologs of the major *Drosophila α1 *isoform, expressed in somatic cells and the testis [16,17], and are most conserved alpha tubulins (Table 2). The second clade consists in the minor *α2 *isoforms (0.95) expressed in *Drosophila *visceral mesoderm and testis cyst cells [8,16,17]. The *α2 *isoform is absent in *Acyrthosiphon, Nasonia, Anopheles*, and *D. persimilis *and *D. psuedoobscura*. The *α3 *clade (1.0) are orthologs of the *Bmα3 *testis-specific isoforms [35]. The *alpha 3 *clade falls within the *α1 *tubulins, indicating its origin in an *α1 *duplication event in a Coleopteran/Lepidopteran/Dipteran ancestor that was lost in Dipterans. The fourth clade consists in the *α4 *isoforms (0.97), which is ovary-specific in *Dm *[16,17], with losses in *Ap*, *Nv*, *Bm*, and *Ae*.

Duplications of the major *α1 *isoform occurred in a number of insect lineages *Acyrthosiphon *(*Apα1a-c*: (0.97, 0.94), *Hymenoptera [Apis *(*Amα1a, b*) and *Nasonia *(*Nvα1a-c*): (0.96)]. While not supported in the zero-fold degeneracy codon tree, a three codon NJ tree (not shown) provides strong support for independent *α1 *duplication events in *Aedes α1a, b*:(99), *Anopheles Agα1a, b*:(98) and *melanogaster *subgroup *Drosophila Ds, Dc, Dy, De *and *Dmα1a, b*: (99) ancestors respectively.

### Sequence distances and carboxy terminus tail sequences

The greatest pairwise distances between any two beta and alpha tubulin protein sequences are 0.464 (*Tc*β4 vs. *Nv*β1) and 0.700 (*Ag*α2 vs. *Am*α4) respectively, which reveals that a wide range of amino acid sequence identities are capable of supporting microtubule assembly per se (Tables 1, 2). Within this overall diversity, orthologs sequence identities are highly conserved, with average pairwise distances among orthologs less than 0.011 for the major α1 and β1 isoforms, and less than 0.140 for the α2, β2 (excluding divergent duplication products), β3, and β4 minor isoforms. This suggests that tubulin evolution is not constrained by microtubule assembly, but by cell-type specific functions. The α3 testis and α4 ovary-specific isoforms, and testis *β2 *tubulin duplication products are exceptions, with average pairwise distances >0.23, >0.55 and >0.46 respectively. These reproductive isoforms are the most variable tubulins in sequence. The major isoforms are the most conserved, and there is not a single non-synonymous substitution among the *Drosophila β1 *and *α1 *tubulins.

Over 50% of the residues that distinguish tubulin paralogs from each other are found in the carboxy terminus tails (CTT). The CTT lies on the surface of the tubulin protein, being free from protein folding permits its greater variability [29]. Because it serves as a site for many tubulin post-translational modifications (PTMs) and for binding tubulin interacting proteins, it is an important mediator of tubulin function.

### PTM motifs

Tubulins can undergo numerous post-translational modifications, which occur on specific tubulin sequence motifs. Polyglutamylation, polyglycylation, detyrosination, and acetylation are modifications associated with stable MT arrays and motile axonemes [40,41], phosphorylated MTs are excluded from mitotic arrays [42], palmitoylation may contribute to the membrane localization of tubulin [43], and interactions with +TIPS (plus-end tracking proteins) have been shown to be inhibited by detyrosination [44].

PTMs that occur on the beta tubulins are polyglutamylation and polyglycylation on a subset of glutamic acid residues on the CTTs [38-40], and phosphorylation of a conserved Ser^178 ^[42]; these sites are found on most beta tubulins (Table 1). The same PTMs that occur on beta tubulins also occur on alpha tubulins, in addition, alpha tubulins can undergo acetylation of Lys^40^, detyrosination of the 3' terminal Tyr, and palmitoylation of Cys^387 ^[[43-45]; Table 2]. Alpha tubulins show much more variation in PTM motifs than beta tubulins, both between paralogs and among orthologs, indicating alpha tubulins more typically underlie PTM-based microtubule specializations. Note that presence of a PTM sequence motif is necessary, but not sufficient for a PTM to occur; regulation of tubulin modifying proteins will play an important role in PTM-based cell type specializations.

### Selection Tests

Selection tests performed between *Drosophila *tubulin orthologs and recent gene duplication products (*Bmβ1a, b; Tcβ2a, b; Nvβ2a-c; Apβ2a-d; Aeβ4a, b; Aeα1a, b; Agα1a, b; Amα1a, b; Nvα1a-c; Apα1a-c*) find strong purifying selection; dN/dS ranges from 0.070 to 0.000 in all pairwise comparisons with a high degree of statistical significance (p = 0.00) (Additional File 2). The last 60 nucleotides that comprise the CTT were also tested for mode of selection based on their different role in tubulin folding and function [29]. There is evidence of positive selection acting on the CTT of insect *β2 *duplication products, and on some *D. spp. α4 *tubulins (p < 0.01), however, the small alignment length after gap removal requires this result be taken with caution.

### Rate Tests

The branch lengths in the genealogy indicate clear differences in tubulin evolutionary dynamics not captured by tests of selection, such that we used Tajima's rate test to identify substitution rate difference among tubulin proteins.

#### Rates tests between ancient tubulin paralogs

The major and minor insect isoforms have their origin in duplication events on major tubulin ancestors. The lack of overlap in major and minor isoform expression domains in *Bombyx *and *Drosophila*, the two insects in which expression data is available, indicates subfunctionalization followed these duplication events, resulting in the minor isoforms. Amino acid rate tests find that minor tubulins evolve more rapidly than major α1 and β1 tubulins (Table 3); divergence in sequence followed duplication and subfunctionalization.

**Table 3:** Rate tests between major and minor tubulin paralogs.

Major isoform	Minor isoform	Rate test (mean Chi-Sq. +/- Sdv)	
Insect β1	Insect β2	5.12 +/- 3.81	n = 9

Insect β1	Insect β3	11.85 +/- 4.33	n = 9

Insect β1	Insect β4	21.34 +/- 21.15	n = 6

Insect α1	Insect α2	10.66 +/- 17.34	n = 6

Insect α1	Insect α3	71.19 +/- 9.91	n = 2

Insect α1	Insect α4	97.87 +/- 62.34	n = 4

Protein evolutionary rates were compared between major and minor tubulin paralogs resulting from ancient duplication events predating separation of insect orders, using *Ha *major alpha and beta tubulins as outgroups. The average and standard deviation of chi-square values for pairwise rate tests are presented (eg. Insect β1 vs. Insect β2 = (Chi-sq. *Dm*β1 vs. *Dm*β2 + Chi-sq. *Ag*β1 vs. *Ag*β2 + Chi-sq. *Ae*β1 vs. *Ag*β2...)/9). In taxa with multiple copies of an isoform, rate tests are performed using the conserved isoform. The only *Drosophila *species included is *Dm*, to avoid a Dipteran skew in the results. Chi-Sq. values > 3.8 have a probability of p < 0.05; Chi-Sq. values > 5.2, p < 0.01.

#### Rate tests between tubulin orthologs

Tubulin major isoform orthologs vary in the amount of pleiotropy in their function, which may have rate effects. All insects have a *β2 and β3 *isoforms, but some do not have *β4, α2, α3 *and/or *α4 *isoforms. In these taxa the major isoform takes on this minor isoform function, resulting in different amounts of pleiotropy for the major isoforms. For example, the major *Nvα1 *isoform supports both somatic, testis, and ovary alpha tubulin function, vs. the *Tcα1 *isoform that only supports somatic function. These differences in pleiotropy do not affect their rates of evolution, all of the insect major α1 and β1 proteins evolve at the same rate respectively (Table 4).

**Table 4:** Rate tests on tubulin orthologs.

Beta 1	Mean Chi-Sq. Vs. Orthologs (n = 8)	Beta 2	Mean Chi-Sq. Vs. Orthologs (n = 8)	Beta 3	Mean Chi-Sq. Vs. Orthologs (n = 8)	Beta 4	Mean Chi-Sq. Vs. Orthologs (n = 4)
*Dm*β1	1.08 +/- 0.91	*Dm*β2	1.02 +/- 2.09	*Dm*β3	0.71 +/- 0.70	*Dm*β4	1.62 +/- 1.72

*Ae*β1	1.49 +/- 1.51	*Ae*β2	1.47 +/- 3.28	*Ae*β3	1.61 +/- 1.46	*Ae*β4a	1.29 +/- 0.44

*Ag*β1	0.96 +/- 0.98	*Ag*β2	5.54 +/- 2.11	*Ag*β3	1.19 +/- 1.20	*Ag*β4	1.68 +/- 1.65

*Bm*β1a	1.65 +/- 1.05	*Bm*β2	1.01 +/- 1.42	*Bm*β3	2.31 +/- 1.89	*Ap*β4	2.53 +/- 1.49

*Tc*β1	1.19 +/- 1.58	*Tc*β2a	1.56 +/- 2.47	*Tc*β3	2.82 +/- 1.75	*Ph*β4	29.84 +/-24.42

*Am*β1	0.97 +/- 0.98	*Am*β2a	1.50 +/- 1.94	*Am*β3	1.44 +/- 1.22		

*Nv*β1	0.74 +/- 1.09	*Nv*β2a	1.25 +/- 2.06	*Nv*β3	1.48 +/- 1.35		

*Ap*β1	2.18 +/- 2.17	*Ap*β2a	0.57 +/- 0.93	*Ap*β3	2.69 +/- 1.81		

*Ph*β1	0.51 +/- 0.44	*Ph*β2a	0.18 +/- 0.16	*Ph*β3	5.91 +/- 3.03		

**Alpha 1**	**Mean Chi-Sq. Vs. Orthologs** **(n = 8)**	**Alpha 2**	**Mean Chi-Sq. Vs. Orthologs** (n = 4*)	**Alpha 3**	**Mean Chi-Sq. Vs. Orthologs** **(n = 1)**	**Alpha 4**	**Mean Chi-Sq. Vs. Orthologs** **(n = 5)**

*Dm*α1a	0.29 +/- 0.23	*Dm*α2	2.37 +/- 3.34	*Bm*α3	0.56	*Dm*α4	7.29 +/- 10.41

*Ae*α1a	1.42 +/- 0.98	*Ae*α2	4.76 +/- 0.60	*Tc*α3	0.56	*Ag*α4a	5.46 +/- 5.83

*Ag*α1a	0.48 +/- 0.42	*Bm*α2	1.69 +/- 2.91			*Tc*α4	13.31 +/- 18.91

*Bm*α1	0.74 +/- 1.05	*Tc*α2	1.51 +/- 2.31			*Am*α4	22.17 +/- 11.79

*Tc*α1	0.43 +/- 0.71	*Am*α2	56.30 +/- 6.55				

*Am*α1a	0.45 +/- 0.69	*Ph*α2	1.63 +/- 1.72				

*Nv*α1a	0.29 +/- 0.25						

*Ap*α1a	0.44 +/- 0.44						

*Ph*α1a	0.83 +/- 1.05						

Protein evolutionary rates were compared among tubulin orthologs. The average +/- Sdv of chi-square values between each tubulin isoform and its insect orthologs is presented (eg. *Dm*β1 = (Chi-sq. *Dm*β1 vs. *Ag*β1 + Chi-sq. *Dm*β1 vs. *Ae*β1 +...)/8), using *Pediculus humanus corporis *orthologs as outgroups; *Pediculus *rates tested with *Acyrthosiphon pisum *outgroups. In taxa with multiple copies of an isoform, the conserved isoform is used in the rate test. *The highly divergent *Am*α2 isoform is an outlier, and was removed from the analysis. Chi-Sq. values > 3.8 have a probability of p < 0.05; Chi-Sq. values > 5.2, p < 0.01.

The tubulin minor orthologs in general evolve at the same rate (Table 4), with the exception of the α4 ovary-specific proteins, and divergent testis β2 duplication products (Table 5).

**Table 5:** Rate tests on tubulin gene duplication products.

Product 1	Product 2	Rate test (Chi-Sq., p)	
*Bm*β1a	*Bm*β1b	1.80	p = 0.179

*Tc*β2a	*Tc*β2b	79.45	p = 0.000

*Am*β2a	*Am*β2b	5.40	p = 0.020

*Nv*β2a	*Nv*β2b	61.81	p = 0.000

*Nv*β2a	*Nv*β2c	76.17	p = 0.000

*Ap*β2a	*Ap*β2b	0.14	p = 0.705

*Ap*β2a	*Ap*β2c	0.00	p = 1.00

*Ap*β2a	*Ap*β2d	24.00	p = 0.000

*Ph*β2a	*Ph*β2b	18.96	p = 0.000

*Ae*β4a	*Ae*β4b	11.31	p = 0.001


*Dm*α1a	*Dm*α1b	2.00	p = 0.157

*Ae*α1a	*Ae*α1b	1.00	p = 0.317

*Ag*α1a	*Ag*α1b	0.33	p = 0.563

*Am*α1a	*Am*α1b	0.00	p = 1.000

*Nv*α1a	*Nv*α1b	0.20	p = 0.654

*Nv*α1a	*Nv*α1c	1.29	p = 0.256

*Ap*α1a	*Ap*α1b	7.36	p = 0.007

*Ap*α1a	*Ap*α1c	4.00	p = 0.046

*Ag*α4a	*Ag*α4b	10.64	p = 0.001

Protein evolutionary rates are compared between tubulin duplication products that postdate separation of insect orders, using *Pediculus humanus corporis *outgroups. *Apβ2a *is used as the outgroup in *Pediculus *rate tests.

#### Recent duplication events

With respect to more recent duplication events, those post-dating separation of the insect orders, gene duplication did not necessarily result in divergent duplication products. Most recent gene duplications appear in the major *alpha 1 *and minor *beta 2 *clades. Recent duplication events always generated at least one conserved product (evolving at the same rate as its orthologs in taxa that did not experience gene duplication). The second product was also conserved in two of eight minor *β2 *isoform duplication events, and in six of the eight major *α1 *isoform duplications (Table 5).

The *Bmβ1a *and *Bmβ1b *duplication products are undergoing subfunctionalization. Both have wide, but only partially overlapping expression domains; of 16 expression domains tested, they overlap in five, nine are unique to *Bmβ1a*, and two are unique to *Bmβ1b *[35]. Both *Bm*β1a and *Bm*β1b proteins have the same substitution rates as other β1 isoforms (Table 5). The *Dmα1a, b *products of the melanogaster subgroup *α1 *duplication are also undergoing subfunctionalization, but of a different kind. *Dmα1b *it is expressed in the same domain as the *Dmα1a *duplication product, but at much reduced levels [16,17]. These products are evolving at the same, slow rate as the other α1 proteins (Table 5).

#### Co-evolution between alpha and beta tubulin

Co-functional links between alpha and beta tubulin must be relatively strong to be detected in co-evolution between proteins. Nonetheless, Sato's Mirror-Tree test of co-evolution finds that the alpha-beta tubulins in the *Drosophila *α2-β3 dimer co-evolve (Correlation 0.4258, p < 0.01). As there is nothing unusual about the α2-β3 dimer to indicate it is not representative of other tubulins, co-function in the dimer may attenuate rates of tubulin evolution, as change in one tubulin to some extent requires change in the other. Contacts between alpha and beta tubulin along the protofilament and between filaments (inter- and intradimer contacts) are known [46], based on these, none of the α2-β3 co-evolving amino acids are in contact with each other. On the other hand, the CTT residues are sites of PTMs, and co-function in this regard could underlie α2-β3 co-evolution. Evolving amino acids in α2: (first, nucleotide-binding domain: 42, 50, 68, 70, 128; second, taxol-binding domain: 236, 289; third, carboxy-terminus domain: 430, 448, CTT 451-57). Evolving amino acids in β3: (nucleotide-binding domain: 5, 23, 32, 33, 47, 58, 60, 98; taxol-domain: 291; CTT: 442-444.

### Intron evolution

**Figure 4:**
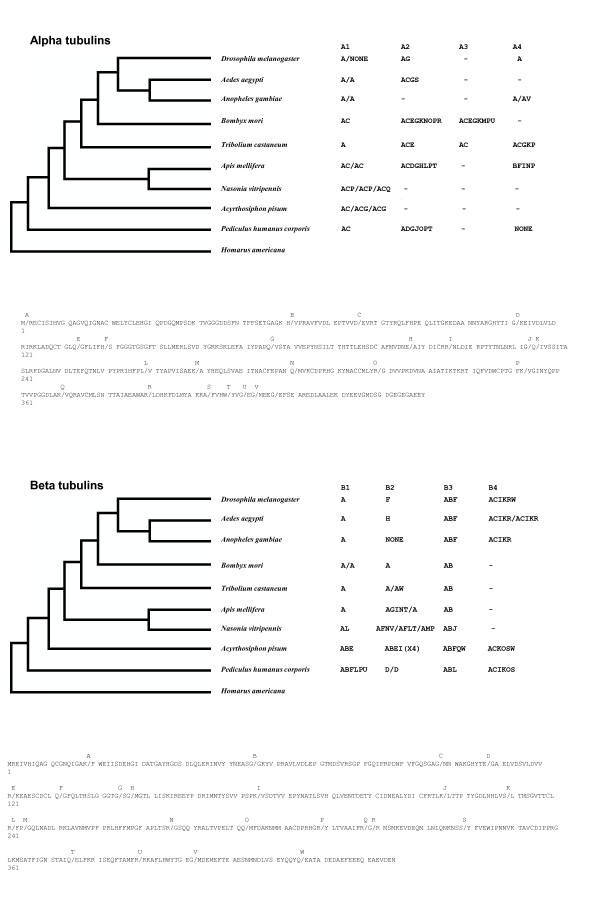
**Beta and alpha tubulin introns**. Beta and alpha tubulin introns are plotted on an insect phylogeny [19], and on *Dm*α1 and *Dm*β1 protein sequences. Introns are labeled A-W and A-V, from the most 5' to most 3' intron found in beta and alpha tubulins. Introns for taxa with multiple copies of an isoform are presented in order, ie. *Nvα1a/Nvα1b/Nvα1c*.

Forty-five different introns are present in alpha and beta tubulins (Fig. 4, Additional Files 3, 4), and their distribution indicates they are very mobile. Given the potential for intron insertion and removal to alter tubulin coding sequence, the abundance and dynamic movement of introns among tubulins is surprising.

One way to assess intron evolution is to plot their presence on an insect phylogeny [19], and assume that introns present in more than one isoform were present in the major isoform ancestor to all insect tubulins (Fig. 4). Twenty-three beta tubulin introns were identified. The DGIPRST and U introns each are unique gains in the beta tubulins in which they reside. The CJMP and Q introns are common to insect *β4*, with losses of MP and Q in Dipteran *β4*. The remaining 9 introns, ABEFHKLNO, are present in >1 beta tubulin isoform, suggesting they were present in the major beta ancestor to the insect tubulins. However, except for the A intron, the number of independent losses required for this explanation seems sufficiently large to argue against it. Rather, a combination of independent gains, losses, and lateral transfer via recombination between paralogs, for example in EN and P, likely explains their representation.

Twenty-two alpha tubulin introns were identified. Eleven introns, BFHIKLPQRTU, are unique gains in the alpha tubulins in which they reside. Three introns, DM and S, are found only in *α2 *isoforms. The remaining seven introns, ACEGJN and O, are present in >1 isoform. Again, except for the A intron, their presence/absence patterns require too many independent losses to assume they were present in the major alpha ancestor to the insect tubulins.

An important mechanism of intron loss is through recombination with reverse transcribed tubulin mRNA sequences [47,48]. The most 5' introns are in general the most conserved in both alpha and beta tubulins, consistent with this mechanism. Mechanisms of intron insertion remain largely a mystery [49,50]. Introns found in two paralogs in the same species, such as the beta tubulin EN and P introns and the alpha tubulin E intron, indicate horizontal transfer of the intron through gene conversion or double recombination between paralogs [50].

The majority of introns are found at sites that are highly conserved across all tubulins (Additional Files 3, 4), suggesting intron insertion must accommodate sequence requirements of the protein, rather than visa versa. There are preferences for certain amino acids bracketing insertion sites, for example, glycine resides bracket 16% of intron splice sites, more than twice their frequency in insect tubulins. There are a few observations indicating intron insertion either altered coding sequence or unusual coding sequence facilitated intron insertion. Five of the 20 unique introns (found only in a single tubulin) are correlated with unusual amino acid identities at the insertion sites, the *Amα4 *F and I introns, the *Amα2 *H and G introns, and the *Phβ2b *D intron.

Tubulin introns are typically large and could therefore be prone to splicing mistakes [51,52]. While ortholog-specific introns are likely promoted by selection, by virtue of the intron insertion event coinciding with the duplication event that led to the isoform, the evolutionary benefit of unique introns in established isoforms requires an explanation. Alternate splicing is not known in tubulin, and thus does not provide a utility to introns. However, regulatory sequences are known to reside in tubulin introns [7,53,54], and if present provide a plausible benefit for intron insertion into established isoforms. Large introns could benefit tubulins, as they reduce Hill-Robertson interference within genes [55].

There is evidence that movement from phase 0 to 1 or 2 accompanies the evolution of old introns as splice signals move from the exon to the intron, while disrupting coding sequence might bias recent introns to phase 0 insertion [56]. There is no association between intron phase and age, 6/11 conserved (old) introns and 7/20 unique (new) introns are phase zero, and except in the few previously mentioned cases, intron insertion regardless of phase does not affect tubulin coding sequence. Intron splice sites tend to remain conserved over time; only the beta tubulin Q and R introns and alpha J and K introns are possibly the same intron undergoing splice site movement.

### Evolution of Drosophila beta 2 tubulin cis-regulation

**Figure 5:**
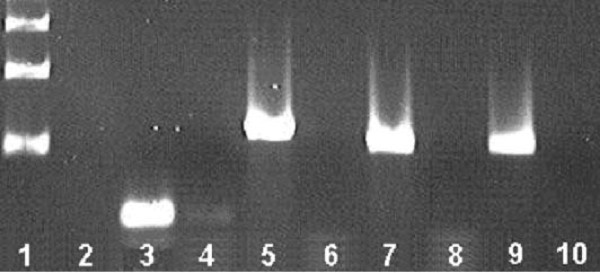
**β2 mRNA expression in *Drosophila *testis tissue**. Testis expression of *Beta 2 *message was tested in *D. ananassae, D. willistoni, D. persimilis*, and *D. psuedoobscura*, species in which the B2UE1 testis cis-regulatory sequence was not identifiable (Table 6). All were found to express *Beta 2 *in the testis, indicating compensatory mutation in testis cis-regulation has occurred to maintain their testis expression. Lane Numbers (L to R) 1. Ladder (bottom most band = 500 bp). 2. blank 3. *D. willistoni *RT-PCR 4. *D. willistoni *Taq PCR 5. *D. ananassae *RT-PCR 6. *D. ananassae *Taq PCR 7. *D. persimilis *RT-PCR 8. *D. persimilis *Taq PCR 9. *D. pseudoobscura *RT-PCR 10. *D. pseudoobscura *Taq PCR.

*D. melanogaster β2 *regulatory elements are conserved in some Diptera, in others they are not found (Table 6). In a subset of these species (*D. willistoni, D. ananassae, D. persimilis, D. pseudoobscura*) testis expression of the *β2 *gene was tested, and confirmed through RT-PCR (Fig. 5). The maintenance of testis expression in view of the loss of previously identified testis regulatory elements indicates that compensatory evolution has occurred in their *β2 *cis-regulation. While the basis of this compensation is not known, it may be more complex than simple re-positioning of regulatory elements, as this would have been identified through our analysis.

**Table 6:** *Drosophila *β2 cis-regulatory sequences.

Species	B2UE1	B2UE2	Inr	B2DE1	ATG
*D. melanogaster*	(-51) ATCGTAGTAGCCTA	(-32) GAACAT	(-3) TTCAGTT	(+51) AAAATTATACGTTTAAAT	+172
*D. ananassae*	(-103) ACCCGAGTATCGTT	(-57) GAACAG	(-3) TCCACCT	(+47) AAAATTGTACGTTAAAAA	+212
*D. erecta*	(-51) ATCGTAGTAGCCCA	(-32) GAACAT	(-3) TTCAGTC	(+51) AAAATTATACGTTTAAAT	+211
*D. grimshawi*	(-328) ATCAGAATTGTTCG	(-256) GAATAT	(-3) CTCATTC	(+49) AAAATTAAACGTGAAAAA	+155
*D. mojavensis*	(-51) ATCCCAGTAGTTCC	(-32) GTACAT	(-3) CTCATTC	(+48) AAAATTATACGTTAAAAT	+187
*D. persimilis*	(-304) CATGTAGAGACCCA	(-55) GAACAA	(-3) CTCATTC	(+43) TAACTTAAAAAATTCATT	+196
*D. pseudoobscura*	(-304) CATGTAGAGACCCA	(-55) GAACAA	(-3) CTCATTC	(+43) TAACTTAAAAAATTCATT	+195
*D. sechellia*	(-51) ATCGCAGTAGCCTA	(-32) GAACAT	(-3) TTCAGTT	(+51) AAAATTATACGTTTAAAT	+169
*D. simulans*	(-51) ATCGCAGTAGCCTA	(-32) GAACAT	(-3) TTCAGTT	(+51) AAAATTATACGTTTAAAT	+165
*D. virilis*	(-51) ATCGAAGTAGTCTA	(-32) GGACAT	(-3) CTCATTC	(+48) AAAATTATACGTAAAAAT	+169
*D. willistoni*	(-189) ATCGAAGAATATTA	(-165) GAACAT	-(3) TCCAGCT	(+46) AAAATTATTCGTACAAAA	+205
*D. yakuba*	(-51) ATCGTAGTAGCCCA	(-32) GAATAT	(-3) CTCAGTC	(+51) AAAATTATACGTTTAAAT	+155
*Anopheles gambiae*	(-79) GCCGTACGTGCCGG	(-52) GAACCT	(-3) TCCATTC	(+45) AAACTAGAAATTTGTGTA	+188

The cis-regulatory elements required for *β2 *tubulin expression in the testis at appropriate levels have been identified in *D. melanogaster*; these sequences (when identifiable) are presented for other *Drosophila *species. Numerical positions indicating sequence position relative to transcription start site (= +1). The B2UE1 element is required for testis-specific gene expression, the B2UE2 and B2DE1 elements for proper expression levels, the Inr element is part of the *β2 *core promotor, and ATG is the start of *β2 *coding sequence.

## Discussion

Tubulins have stringent structure/function relationships, indicated by strong purifying selection, the loss of many gene duplication products, alpha-beta co-evolution in the tubulin dimer, and compensatory evolution in *beta 2 tubulin *cis-regulation. Gene duplication, subfunctionalization in expression domain, and divergence, particularly in CTT sequences has resulted in the specialized, minor tissue-specific insect isoforms. Conservation of ortholog sequence identities and expression patterns in *Bm *and *Dm *suggests ortholog function might be ancient and largely shared among insects, having been established in their common ancestor. The exception to conservation is in the *α3*, *α4*, and *β2b, c *isoforms. The great sequence variability in these reproductive tissue-specific tubulins indicates species-specific function, and illustrates that even a highly conserved protein family can participate in the adaptive process and respond to sexual selection [57,58].

Pairwise distances between tubulin paralogs reveals a wide variety of tubulin sequences are able to generate an MT array, such that the slow rate of ortholog evolution does not result from a lack of sequences able to generate microtubule arrays. Furthermore, testis-specific isoforms support a wider diversity of microtubule arrays than do major somatic tubulins, yet show more sequence diversity than major isoforms, moreover, somatic tubulins with reproductive function, like *Nvα1*, do not evolve more slowly than those without. These observations indicate that pleiotropy in microtubule array support does not constrain tubulin evolution; more generally, that support of MT arrays *per se *is not main source of purifying selection on tubulin sequence.

Path-dependence in the order of amino acid change has been proposed as an important constraint in the evolution of beta 2 tubulin residues that participate in an amino acid synergism [1], and may be a general constraint in residues involved in protein folding. This local constraint would result in purifying selection, yet allow for variation among paralogs to build over time as viable evolutionary pathways are found.

In addition, ortholog conservation may result from support of more subtle, cell-type specific aspects of tubulin function that involve sorting among different MT arrays and the timing of MT array generation. These aspects are mediated by CTT sequences. CTTs do not participate in protein folding, but mediate the tubulin code by providing sequence motifs for PTMs, and by mediating interactions with tubulin associated proteins. CTT sequences can influence subcellular localization of different MT arrays, interactions with plus-end tracking proteins (+TIPS) that influence dynamic instability, and sites for motor proteins to preferentially bind [41]. CTT variation can provide MT specializations, for example, insects with unusual axonemes show reduced levels of both polyglutamylation and polyglycylation [39,59]. Conversely, avoiding unusual MT arrays may contribute to the conservation of major somatic isoform CTTs, which need to function in "normal" MT arrays across a diversity of cell types.

### Role of gene duplication in tubulin evolution

Insight into the ancient duplication events that generated the major and minor insect tubulins can be found in more recent duplication events. Duplication events that post-date separation of the insect orders are unevenly distributed among tubulins, with most occurring on *alpha 1 *and *beta 2 *templates. Many duplication products are lost, likely because they are deleterious; tubulins are incorporated into MT arrays as a function of cellular concentration, thus diverging duplication products have the potential to poison existing MT arrays, resulting in selection against them. This argues against the classical model of duplication and divergence [60], as without positive selection, in most cases a duplicate gene would be lost before finding novel function. The duplication-degeneration-complementation model [61] proposes that degenerative mutations may accumulate in each duplication product, resulting in subfunctionalization. This alleviates the need for positive selection to operate in order to maintain duplicated genes. Subfunctionalization may explain why major and minor duplication products differ in their fate: 8/10 minor isoform duplications result in a rapidly-evolving and a conserved product, while 7/9 major isoform duplications result in two equally conserved products. Minor isoform expression is already confined to narrow expression domain, removing cis-evolution (and subfunctionalization) as a potential prerequisite for their retention and diversification.

### Roles of beta and alpha tubulin in specialized MT arrays

Beta tubulins vary little in PTM sites, such that functional variation among them resides in the tubulin modifying protein composition of a cell, not the beta tubulin. Conversely, the alpha tubulins both experience a wider range of PTMs, and show more variation in PTM sequence motifs, and therefore might be more fundamental in mediating the tubulin code.

In addition to this role in PTMs, alpha tubulin may also have the greater potential to specialize in function, thereby playing a role in adaptation, because it seems more dispensable. Only one alpha tubulin, *α1*, is present in every insect order, as compared to three beta tubulins, *β1, β2, β3*. Loss of the *α2 *gene in *D. persimilis *and *D. pseudoobscura *correlates with short sperm and oval testis morphology unique in their genus. Alpha tubulins also show a great amount of standing variation in "unevolved" *α1 *duplication products that have the potential to participate in the adaptive process.

On the other hand, Tuszynski in his review of vertebrate tubulins [15] suggests the beta tubulin component may be more associated with MT array specializations, the number of beta minor isoforms is greater in most vertebrates than alpha minor isoforms, and more beta minor isoforms co-function with a major, "vanilla" alpha major isoform than visa versa. This seems to also hold largely true for the insects, as many insect species express only the major alpha isoform, but multiple beta isoforms, while all but *Bombyx *have 4 distinct beta isoforms.

Co-evolution in the alpha/beta tubulin dimer reveals that both the alpha and the beta component are associated with MT array specializations. Although the particular co-evolving residues do not suggest a clear structural basis for co-evolution in α2 and β3, a basis for co-evolution in mediating the tubulin code is quite possible in co-evolving CTT residues. It has been shown that either the alpha or beta tubulin CTT can serve as the donor of a PTM site [62], an interrelationship that provides a mechanism for PTM-based alpha/beta co-evolution. More generally, specialized MT arrays can be mediated by either dimer component: the specialized *Dm*β2 functions with the major *Dm*α1 tubulin in *Drosophila *axoneme, the major *Dm*β1 functions with a specialized *Dm*α4 in the ovary, the minor α3/β2 isoforms support the *Bombyx *motile axoneme, and the minor α2/β3 isoforms support the *Drosophila *testis cyst cell in which the gigantic spermtails distinctive of *Drosophila *are generated. Given this variety of relationships between alpha and beta tubulin and MT specializations, which component provides specialization may well be due to evolutionary chance.

## Conclusion

Gene duplication alone is not sufficient for tubulin evolution. Most gene duplication products do not survive, whether through direct elimination or pseudogenization. Those that do survive evolve only when followed by a narrowing in the expression domain. Given the number of rare events that must occur (viable gene duplication, subfunctionalization, path-dependent evolution of coding sequence under purifying selection) to result in a novel tubulin, their slow rate of evolution seems best explained by limitations on such opportunities, especially given the great variation in tubulin proteins found in distance comparisons. Such opportunities do not seem exhausted given vertebrates express 7-8 alpha and beta tubulins; the rapid evolution of the reproductive tubulins also reveals a use for divergent tubulins. It also provides chance a fundamental role in shaping tubulin evolution it terms of when these events occur, providing an allele for selection to choose from. "Evidence" of this role being real is seen in the odd distribution of isoforms, duplication events, and divergent duplication products, and which component of the dimer, alpha, beta, or both, underlies a microtubule specialization.

One important exception is in reproductive tissue-specific isoforms, which show a large amount of variation potentially capable of responding to sexual selection, a fundamental force in insect evolution. Reproductive isoforms have the fewest PTMs, and the most unusual spermtail axonemes are accompanied by reduction of PTM modifications [59]. Relaxation of the informational aspect of tubulin function might release tubulins to contribute to specialized testis phenotypes typical of insect evolution. Continued study might show more such relaxations, a form of co-evolution fostering the evolvability of an important gene family.

## Competing interests

The authors declare that they have no competing interests.

## Authors' contributions

MN conceived of the study, carried out the sequence retrieval, determination of intron/exon structure, the Bayesian genealogy, rate tests, and drafted the manuscript. SG carried out ML selection test, contributed to the data analysis, and editing the manuscript. LG performed the testes RNA preparations and RT-PCR for the different *Drosophila *species. All authors read and approved the final manuscript.

## Supplementary Material

Additional file 1 **Insect tubulin sequence accessions**. Insect species, tubulin isoform, and accessions are presented. Accessions beginning with gi derive from Flybase blast searches, other accessions from Genbank. Click here for file

Additional file 2 **Likelihood tests of mode of selection acting on tubulin**. Maximum likelihood tests of selection are performed on *Drosophila *tubulins, and genes resulting from recent duplication events (those restricted to a single insect order) show that duplicated genes evolve under purifying selection. The entire coding sequence (beta tubulin nt 1-1422, alpha tubulin nt 1-1422) was analyzed. Analyses were conducted using the Nei-Gojobori method in PAML [30]. Alignment gaps were eliminated by complete deletion. Click here for file

Additional file 3 **Beta Tubulin Intron Features**. Intron representation in insect tubulins, intron phase, length, and splice donor/acceptor sites are presented. The amino acids bracketing the splice site, whether the spice site is within unique 5' and 3' codon sequence, and whether tubulins with the intron share the same sequence is presented, to indicate associations between intron presence/absence and tubulin coding sequence. Key: M = A or C, K = G or T, R = A or G, Y = C or T, W = A or T, S = C or G, V = not T, H = not G. Click here for file

Additional file 4 **Alpha Tubulin Intron Features**. Intron representation in insect tubulins, intron phase, length, and splice donor/acceptor sites are presented. The amino acids bracketing the splice site, whether the spice site is within unique 5' and 3' codon sequence, and whether tubulins with the intron share the same sequence is presented, to indicate associations between intron presence/absence and tubulin coding sequence. Key: M = A or C, K = G or T, R = A or G, Y = C or T, W = A or T, S = C or G, V = not T, H = not G. Click here for file
