# Supplementary material for: Tubulin evolution in insects: gene duplication and subfunctionalization provide specialized isoforms in a functionally constrained gene family
Source: BMC Evol Biol. 2010 Apr 27;10:113. doi: 10.1186/1471-2148-10-113 (PMC2880298; doi:10.1186/1471-2148-10-113)
Supplement: Additional file 1 — Insect tubulin sequence accessions. Insect species, tubulin isoform, and accessions are presented. Accessions beginning with gi derive from Flybase blast searches, other accessions from Genbank. [file 1471-2148-10-113-S1.DOC]

**Additional File 1. Insect tubulin sequence accessions.**

| Species/Gene | Accession Number |
| --- | --- |
| *Acyrthosiphon pisum β1*  *Apβ1* | XM_001951727; GeneID:[100168148](http://www.ncbi.nlm.nih.gov/sites/entrez?db=gene&cmd=Retrieve&dopt=full_report&list_uids=100168148) |
| *Acyrthosiphon pisum β2a*  *Apβ2a* | XM_001945398; GeneID:100163077 |
| *Acyrthosiphon pisum β2b*  *Apβ2b* | GENE ID: 100158910 LOC100158910 |
| *Acyrthosiphon pisum β2c*  *Apβ2c* | GENE ID: 100168298 LOC100168298 |
| *Acyrthosiphon pisum β2d*  *Apβ2d* | GENE ID: 100165276 LOC100165276 |
| *Acyrthosiphon pisum β3*  *Apβ3* | [XM_001943558.1](http://www.ncbi.nlm.nih.gov/nuccore/193606136); GeneID:[100166459](http://www.ncbi.nlm.nih.gov/sites/entrez?db=gene&cmd=Retrieve&dopt=full_report&list_uids=100166459) |
| *Acyrthosiphon pisum β4*  *Apβ4* | XM_001943558; GeneID:100166459 |
| *Aedes aegypti β1*  *Aeβ1* | [gi|78216703|gb|CH477254|CH477254](http://www.ncbi.nlm.nih.gov/entrez/query.fcgi?cmd=Retrieve&db=Nucleotide&list_uids=78216703&dopt=GenBank) |
| *Aedes aegypti β2*  *Aeβ2* | [gi|78216038|gb|CH477401|CH477401](http://www.ncbi.nlm.nih.gov/entrez/query.fcgi?cmd=Retrieve&db=Nucleotide&list_uids=78216038&dopt=GenBank) |
| *Aedes aegypti β3*  *Aeβ3* | [gi|78216703|gb|CH477254|CH477254](http://www.ncbi.nlm.nih.gov/entrez/query.fcgi?cmd=Retrieve&db=Nucleotide&list_uids=78216703&dopt=GenBank) |
| *Aedes aegypti β4*a  *Aeβ4a* | [gi|78215417|gb|CH477325|CH477325](http://www.ncbi.nlm.nih.gov/entrez/query.fcgi?cmd=Retrieve&db=Nucleotide&list_uids=78215417&dopt=GenBank) |
| *Aedes aegypti β4*b  *Aeβ4b* | [gi|78215417|gb|CH477325|CH477325](http://www.ncbi.nlm.nih.gov/entrez/query.fcgi?cmd=Retrieve&db=Nucleotide&list_uids=78215417&dopt=GenBank) |
| *Anopheles gambiae β1*  *Agβ1* | [gi|19611779|gb|AAAB01008823|AAAB01008823](http://www.ncbi.nlm.nih.gov/entrez/query.fcgi?cmd=Retrieve&db=Nucleotide&list_uids=19611779&dopt=GenBank) |
| *Anopheles gambiae β2*  *Agβ2* | [gb|AAAB01008944.1|](http://www.ncbi.nlm.nih.gov/entrez/query.fcgi?cmd=Retrieve&db=Nucleotide&list_uids=19612093&dopt=GenBank) Anopheles gambiae str. PEST chromosome 3R CRA_x9P1GAV5C5F  EAA10161 (mRNA) |
| *Anopheles gambiae β3*  *Agβ3* | [gb|AAAB01008933.1|](http://www.ncbi.nlm.nih.gov/entrez/query.fcgi?cmd=Retrieve&db=Nucleotide&list_uids=19612062&dopt=GenBank) Anopheles gambiae str. PEST chromosome 3L CRA_x9P1GAV5BYM |
| *Anopheles gambiae β4*  *Agβ4* | [gb|AAAB01008810.1|](http://www.ncbi.nlm.nih.gov/entrez/query.fcgi?cmd=Retrieve&db=Nucleotide&list_uids=19611747&dopt=GenBank) Anopheles gambiae str. PEST chromosome 2L CRA_x9P1GAV5943 |
| *Apis mellifera β1*  *Amβ1* | [ref|NC_007073.1|NC_007073](http://www.ncbi.nlm.nih.gov/entrez/query.fcgi?cmd=Retrieve&db=Nucleotide&list_uids=66830420&dopt=GenBank)  Xm 392313 (mRNA) |
| *Apis mellifera β2*  *Amβ2* | GENE ID: 408782 LOC408782 |
| *Apis mellifera β2b*  *Amβ2* | GENE ID: 410996 LOC410996 |
| *Apis mellifera β3*  *Amβ3* | GENE ID: 410994 LOC410994 |
| *Bombyx mori β1*  *Bmβ1* | [gi|56197530|gb|CH381315|CH381315](http://www.ncbi.nlm.nih.gov/entrez/query.fcgi?cmd=Retrieve&db=Nucleotide&list_uids=56197530&dopt=GenBank)  [AB072307](http://www.ncbi.nlm.nih.gov/entrez/viewer.fcgi?db=nucleotide&val=19773425) (mRNA) |
| *Bombyx mori β2*  *Bmβ2* | [gi|56198717|gb|CH380128|CH380128](http://www.ncbi.nlm.nih.gov/entrez/query.fcgi?cmd=Retrieve&db=Nucleotide&list_uids=56198717&dopt=GenBank)  [AB072308](http://www.ncbi.nlm.nih.gov/entrez/viewer.fcgi?db=nucleotide&val=19773427) (mRNA) |
| *Bombyx mori β3*  *Bmβ3* | [gi|56189641|gb|CH389144|CH389144](http://www.ncbi.nlm.nih.gov/entrez/query.fcgi?cmd=Retrieve&db=Nucleotide&list_uids=56189641&dopt=GenBank)  [AB072309](http://www.ncbi.nlm.nih.gov/entrez/viewer.fcgi?db=nucleotide&val=19773429) (mRNA) |
| *Bombyx mori β4*  *Bmβ4* | [gi|56189425|gb|CH389360|CH389360](http://www.ncbi.nlm.nih.gov/entrez/query.fcgi?cmd=Retrieve&db=Nucleotide&list_uids=56189425&dopt=GenBank)  [AB072310](http://www.ncbi.nlm.nih.gov/entrez/viewer.fcgi?db=nucleotide&val=19773431) (mRNA) |
| *Drosophila melanogaster*  56D  *Dmβ1* | [gnl|dmel|2R](http://flybase.bio.indiana.edu/blast/getSequence.html?jobHash=e5093e3d5befd0b56f71f080cfc39318&seqId=gnl|dmel|2R) type=chromosome; loc=2R:1..20766785; ID=2R; release=r4.3  NM_079071 (mRNA) |
| *Drosophila melanogaster*  85D  *Dmβ2* | [gnl|dmel|3R](http://flybase.bio.indiana.edu/blast/getSequence.html?jobHash=bc93517b269232fa9152ffdcf15eb20b&seqId=gnl|dmel|3R) type=chromosome; loc=3R:1..27905053; ID=3R; release=r4.3  NM_079566 (mRNA) |
| *Drosophila melanogaster*  60D  *Dmβ3* | [gnl|dmel|2R](http://flybase.bio.indiana.edu/blast/getSequence.html?jobHash=5c9edcc74bc822b8bff410e62be387d1&seqId=gnl|dmel|2R) type=chromosome; loc=2R:1..20766785; ID=2R; release=r4.3  NM_079118 (mRNA) |
| *Drosophila melanogaster*  97EF  *Dmβ4* | [gnl|dmel|3R](http://flybase.bio.indiana.edu/blast/getSequence.html?jobHash=5b334c8569c6061454d8799147176ed4&seqId=gnl|dmel|3R) type=chromosome; loc=3R:1..27905053; ID=3R; release=r4.3  [gi|21428639|gb|AY118611.1|](http://www.ncbi.nlm.nih.gov/entrez/query.fcgi?cmd=Retrieve&db=Nucleotide&list_uids=21428639&dopt=GenBank) (mRNA) |
| *Drosophila ananassae* 56D  *Daβ1* | [gi|91853191|gb|AAPP01018714|AAPP01018714](http://www.ncbi.nlm.nih.gov/entrez/query.fcgi?cmd=Retrieve&db=Nucleotide&list_uids=91853191&dopt=GenBank) |
| *Drosophila ananassae* 85D  *Daβ2* | [gi|91850032|gb|AAPP01019523|AAPP01019523](http://www.ncbi.nlm.nih.gov/entrez/query.fcgi?cmd=Retrieve&db=Nucleotide&list_uids=91850032&dopt=GenBank) |
| *Drosophila ananassae* 60D  *Daβ3* | [gi|91852573|gb|AAPP01018786|AAPP01018786](http://www.ncbi.nlm.nih.gov/entrez/query.fcgi?cmd=Retrieve&db=Nucleotide&list_uids=91852573&dopt=GenBank) |
| *Drosophila ananassae* 97EF  *Daβ4* | [gi|91849322|gb|AAPP01019624|AAPP01019624](http://www.ncbi.nlm.nih.gov/entrez/query.fcgi?cmd=Retrieve&db=Nucleotide&list_uids=91849322&dopt=GenBank) |
| *Drosophila erecta* 56D  *Deβ1* | [gi|91889685|gb|AAPQ01007044|AAPQ01007044](http://www.ncbi.nlm.nih.gov/entrez/query.fcgi?cmd=Retrieve&db=Nucleotide&list_uids=91889685&dopt=GenBank) |
| *Drosophila erecta* 85D  *Deβ2* | [gi|91890260|gb|AAPQ01006469|AAPQ01006469](http://www.ncbi.nlm.nih.gov/entrez/query.fcgi?cmd=Retrieve&db=Nucleotide&list_uids=91890260&dopt=GenBank) |
| *Drosophila erecta* 60D  *Deβ3* | [gi|91889647|gb|AAPQ01007082|AAPQ01007082](http://www.ncbi.nlm.nih.gov/entrez/query.fcgi?cmd=Retrieve&db=Nucleotide&list_uids=91889647&dopt=GenBank) |
| *Drosophila erecta* 97EF  *Deβ4* | [gi|91889931|gb|AAPQ01006798|AAPQ01006798](http://www.ncbi.nlm.nih.gov/entrez/query.fcgi?cmd=Retrieve&db=Nucleotide&list_uids=91889931&dopt=GenBank) |
| *Drosophila grimshawi* 56D  *Dgβ1* | [gi|91899357|gb|AAPT01021541|AAPT01021541](http://www.ncbi.nlm.nih.gov/entrez/query.fcgi?cmd=Retrieve&db=Nucleotide&list_uids=91899357&dopt=GenBank) |
| *Drosophila grimshawi* 85D  *Dgβ2* | [gi|91901558|gb|AAPT01019340|AAPT01019340](http://www.ncbi.nlm.nih.gov/entrez/query.fcgi?cmd=Retrieve&db=Nucleotide&list_uids=91901558&dopt=GenBank) |
| *Drosophila grimshawi* 60D  *Dgβ3* | [gi|91899434|gb|AAPT01021464|AAPT01021464](http://www.ncbi.nlm.nih.gov/entrez/query.fcgi?cmd=Retrieve&db=Nucleotide&list_uids=91899434&dopt=GenBank) |
| *Drosophila grimshawi* 97EF  *Dgβ4* | [gi|91902121|gb|AAPT01018777|AAPT01018777](http://www.ncbi.nlm.nih.gov/entrez/query.fcgi?cmd=Retrieve&db=Nucleotide&list_uids=91902121&dopt=GenBank) |
| *Drosophila mojavensis* 56D  *Doβ1* | [gi|91922607|gb|AAPU01010176|AAPU01010176](http://www.ncbi.nlm.nih.gov/entrez/query.fcgi?cmd=Retrieve&db=Nucleotide&list_uids=91922607&dopt=GenBank) |
| *Drosophila mojavensis* 85D  *Doβ2* | [gi|91921809|gb|AAPU01010974|AAPU01010974](http://www.ncbi.nlm.nih.gov/entrez/query.fcgi?cmd=Retrieve&db=Nucleotide&list_uids=91921809&dopt=GenBank) |
| *Drosophila mojavensis* 60D  *Doβ3* | [gi|91922508|gb|AAPU01010275|AAPU01010275](http://www.ncbi.nlm.nih.gov/entrez/query.fcgi?cmd=Retrieve&db=Nucleotide&list_uids=91922508&dopt=GenBank) |
| *Drosophila mojavensis* 97EF  *Doβ4* | [gi|91921699|gb|AAPU01011084|AAPU01011084](http://www.ncbi.nlm.nih.gov/entrez/query.fcgi?cmd=Retrieve&db=Nucleotide&list_uids=91921699&dopt=GenBank) |
| *Drosophila persimilis* 56D  *Dpβ1* | [gi|80982439|gb|CH479183|CH479183](http://www.ncbi.nlm.nih.gov/entrez/query.fcgi?cmd=Retrieve&db=Nucleotide&list_uids=80982439&dopt=GenBank) |
| *Drosophila persimilis* 85D  *Dpβ2* | [gi|80982436|gb|CH479186|CH479186](http://www.ncbi.nlm.nih.gov/entrez/query.fcgi?cmd=Retrieve&db=Nucleotide&list_uids=80982436&dopt=GenBank) |
| *Drosophila persimilis* 60D  *Dpβ3* | [gi|80982409|gb|CH479213|CH479213](http://www.ncbi.nlm.nih.gov/entrez/query.fcgi?cmd=Retrieve&db=Nucleotide&list_uids=80982409&dopt=GenBank) |
| *Drosophila persimilis* 97EF  *Dpβ4* | [gi|80982443|gb|CH479179|CH479179](http://www.ncbi.nlm.nih.gov/entrez/query.fcgi?cmd=Retrieve&db=Nucleotide&list_uids=80982443&dopt=GenBank) |
| *Drosophila pseudoobscura* 56D  *Duβ1* | [gnl|dpse|U](http://flybase.bio.indiana.edu/blast/getSequence.html?jobHash=9f5ea5b77dbd9cf31d00a6bc6ef862de&seqId=gnl|dpse|U) type=chromosome; loc=U:1..21508114; ID=U; release=r2.0; species=dpse |
| *Drosophila pseudoobscura* 85D  *Duβ2* | [gnl|dpse|2](http://flybase.bio.indiana.edu/blast/getSequence.html?jobHash=d8ec3c4251b022fdf151656e7e02af2e&seqId=gnl|dpse|2) type=chromosome; loc=2:1..30794189; ID=2; release=r2.0 |
| *Drosophila pseudoobscura* 60D  *Duβ3* | [gnl|dpse|3](http://flybase.bio.indiana.edu/blast/getSequence.html?jobHash=6145b49db805a0fea3ca2d56c6acaab4&seqId=gnl|dpse|3) type=chromosome; loc=3:1..19779522; ID=3; release=r2.0 |
| *Drosophila pseudoobscura* 97EF  *Duβ4* | [gnl|dpse|2](http://flybase.bio.indiana.edu/blast/getSequence.html?jobHash=3ee562c7db52bd3453e7cfcc6c4b7abd&seqId=gnl|dpse|2) type=chromosome; loc=2:1..30794189; ID=2; release=r2.0 |
| *Drosophila sechellia* 56D  *Dcβ1* | [gi|80980799|gb|CH480816|CH480816](http://www.ncbi.nlm.nih.gov/entrez/query.fcgi?cmd=Retrieve&db=Nucleotide&list_uids=80980799&dopt=GenBank) |
| *Drosophila sechellia* 85D  *Dcβ2* | [gi|80980800|gb|CH480815|CH480815](http://www.ncbi.nlm.nih.gov/entrez/query.fcgi?cmd=Retrieve&db=Nucleotide&list_uids=80980800&dopt=GenBank) |
| *Drosophila sechellia* 60D  *Dcβ3* | [gi|80980777|gb|CH480838|CH480838](http://www.ncbi.nlm.nih.gov/entrez/query.fcgi?cmd=Retrieve&db=Nucleotide&list_uids=80980777&dopt=GenBank) |
| *Drosophila sechellia* 97EF  *Dcβ4* | [gi|80980778|gb|CH480837|CH480837](http://www.ncbi.nlm.nih.gov/entrez/query.fcgi?cmd=Retrieve&db=Nucleotide&list_uids=80980778&dopt=GenBank) |
| *Drosophila simulans* 56D  *Dsβ1* | [gi|62998563|gb|CM000164|CM000164](http://www.ncbi.nlm.nih.gov/entrez/query.fcgi?cmd=Retrieve&db=Nucleotide&list_uids=62998563&dopt=GenBank) |
| *Drosophila simulans* 85D  *Dsβ2* | [gi|62998561|gb|CM000166|CM000166](http://www.ncbi.nlm.nih.gov/entrez/query.fcgi?cmd=Retrieve&db=Nucleotide&list_uids=62998561&dopt=GenBank) |
| *Drosophila simulans* 97EF  *Dsβ4* | [gi|62998561|gb|CM000166|CM000166](http://www.ncbi.nlm.nih.gov/entrez/query.fcgi?cmd=Retrieve&db=Nucleotide&list_uids=62998561&dopt=GenBank) |
| *Drosophila virilis* 56D  *Dvβ1* | [gi|91811047|gb|AANI01014866|AANI01014866](http://www.ncbi.nlm.nih.gov/entrez/query.fcgi?cmd=Retrieve&db=Nucleotide&list_uids=91811047&dopt=GenBank) |
| *Drosophila virilis* 85D  *Dvβ2* | [gi|91808807|gb|AANI01017106|AANI01017106](http://www.ncbi.nlm.nih.gov/entrez/query.fcgi?cmd=Retrieve&db=Nucleotide&list_uids=91808807&dopt=GenBank) |
| *Drosophila virilis* 60D  *Dvβ3* | [gi|91811083|gb|AANI01014830|AANI01014830](http://www.ncbi.nlm.nih.gov/entrez/query.fcgi?cmd=Retrieve&db=Nucleotide&list_uids=91811083&dopt=GenBank) |
| *Drosophila virilis* 97EF  *Dvβ4* | [gi|91808708|gb|AANI01017205|AANI01017205](http://www.ncbi.nlm.nih.gov/entrez/query.fcgi?cmd=Retrieve&db=Nucleotide&list_uids=91808708&dopt=GenBank) |
| *Drosophila willistoni* 56D  *Dwβ1* | [gi|93542988|gb|CH713697|CH713697](http://www.ncbi.nlm.nih.gov/entrez/query.fcgi?cmd=Retrieve&db=Nucleotide&list_uids=93542988&dopt=GenBank) |
| *Drosophila willistoni* 85D  *Dwβ2* | [gi|93542084|gb|CH714085|CH714085](http://www.ncbi.nlm.nih.gov/entrez/query.fcgi?cmd=Retrieve&db=Nucleotide&list_uids=93542084&dopt=GenBank) |
| *Drosophila willistoni* 60D  *Dwβ3* | [gi|93542855|gb|CH713741|CH713741](http://www.ncbi.nlm.nih.gov/entrez/query.fcgi?cmd=Retrieve&db=Nucleotide&list_uids=93542855&dopt=GenBank) |
| *Drosophila willistoni* 97EF  *Dwβ4* | [gi|93542007|gb|CH714104|CH714104](http://www.ncbi.nlm.nih.gov/entrez/query.fcgi?cmd=Retrieve&db=Nucleotide&list_uids=93542007&dopt=GenBank) |
| *Drosophila yakuba* 56D  *Dyβ1* | [gi|84681594|gb|AAEU02000463|AAEU02000463](http://www.ncbi.nlm.nih.gov/entrez/query.fcgi?cmd=Retrieve&db=Nucleotide&list_uids=84681594&dopt=GenBank) |
| *Drosophila yakuba* 85D  *Dyβ2* | [gi|84682049|gb|AAEU02000008|AAEU02000008](http://www.ncbi.nlm.nih.gov/entrez/query.fcgi?cmd=Retrieve&db=Nucleotide&list_uids=84682049&dopt=GenBank) |
| *Drosophila yakuba* 60D  *Dyβ3* | [gi|84681656|gb|AAEU02000401|AAEU02000401](http://www.ncbi.nlm.nih.gov/entrez/query.fcgi?cmd=Retrieve&db=Nucleotide&list_uids=84681656&dopt=GenBank) |
| *Drosophila yakuba* 97EF  *Dyβ4* | [gi|84681823|gb|AAEU02000234|AAEU02000234](http://www.ncbi.nlm.nih.gov/entrez/query.fcgi?cmd=Retrieve&db=Nucleotide&list_uids=84681823&dopt=GenBank) |
| *Nasonia vitripennis*  *Nvβ1* | XM_001600804; GeneID:[100116329](http://www.ncbi.nlm.nih.gov/sites/entrez?db=gene&cmd=Retrieve&dopt=full_report&list_uids=100116329) |
| *Nasonia vitripennis*  *Nvβ2a* | XM_001602310; GeneID:[100114292](http://www.ncbi.nlm.nih.gov/sites/entrez?db=gene&cmd=Retrieve&dopt=full_report&list_uids=100114292) |
| *Nasonia vitripennis*  *Nvβ2b* | XM_001600211; GeneID:[100116454](http://www.ncbi.nlm.nih.gov/sites/entrez?db=gene&cmd=Retrieve&dopt=full_report&list_uids=100116454) |
| *Nasonia vitripennis*  *Nvβ2c* | XM_001606240; GeneID:[100113958](http://www.ncbi.nlm.nih.gov/sites/entrez?db=gene&cmd=Retrieve&dopt=full_report&list_uids=100113958) |
| *Nasonia vitripennis*  *Nvβ3* | XM_001603410; GeneID:[100119733](http://www.ncbi.nlm.nih.gov/sites/entrez?db=gene&cmd=Retrieve&dopt=full_report&list_uids=100113958) |
| *Pediculus humanus corporis β1*  *Phβ1* | XM_002426195; GeneID:8230775 |
| *Pediculus humanus corporis β2*  *Phβ2* | [XM_002424436.1](http://www.ncbi.nlm.nih.gov/nuccore/242007303); GeneID:[8234157](http://www.ncbi.nlm.nih.gov/sites/entrez?db=gene&cmd=Retrieve&dopt=full_report&list_uids=8234157) |
| *Pediculus humanus corporis β2*  *Phβ2b* | [XM_002424455.1](http://www.ncbi.nlm.nih.gov/nuccore/242007341); GeneID:[8234176](http://www.ncbi.nlm.nih.gov/sites/entrez?db=gene&cmd=Retrieve&dopt=full_report&list_uids=8234176) |
| *Pediculus humanus corporis β3*  *Phβ3* | XM_002423072; GeneID:8232430 |
| *Pediculus humanus corporis β4*  *Phβ4* | XM_002428220; GeneID:[8233661](http://www.ncbi.nlm.nih.gov/sites/entrez?db=gene&cmd=Retrieve&dopt=full_report&list_uids=8233661) |
| *Tribolium castaneum β1*  *Tcβ1* | GENE ID: 655614 LOC655614 |
| *Tribolium castaneum β2*  *Tcβ2* | [gb|AAJJ01000015.1|](http://www.ncbi.nlm.nih.gov/entrez/query.fcgi?cmd=Retrieve&db=Nucleotide&list_uids=73486632&dopt=GenBank)  Xm 964900 (mRNA) |
| *Tribolium castaneum β2*  *Tcβ2b* | [gb|AAJJ01000659.1|](http://www.ncbi.nlm.nih.gov/entrez/query.fcgi?cmd=Retrieve&db=Nucleotide&list_uids=73485988&dopt=GenBank)  Xm 963221 (mRNA) |
| *Tribolium castaneum β3*  *Tcβ3* | GENE ID: 655694 LOC655694 |
| *Aedes aegypti α1*a  *Aeα1a* | [gi|78216836|gb|CH477994|CH477994](http://www.ncbi.nlm.nih.gov/entrez/query.fcgi?cmd=Retrieve&db=Nucleotide&list_uids=78216836&dopt=GenBank) |
| *Aedes aegypti α1*b  *Aeα1b* | [gi|78216032|gb|CH477400|CH477400](http://www.ncbi.nlm.nih.gov/entrez/query.fcgi?cmd=Retrieve&db=Nucleotide&list_uids=78216032&dopt=GenBank)  DQ440241 (mRNA) |
| *Acyrthosiphon pisum α1a*  *Apα1a* | GENE ID: 100169457 LOC100169457 |
| *Acyrthosiphon pisum α1b*  *Apα1b* | GENE ID: 100168349 LOC100168349 |
| *Acyrthosiphon pisum α1c*  *Apα1c* | GENE ID: 100165839 LOC100165839 |
| *Aedes aegypti α2*  *Aeα2* | [gi|78216666|gb|CH477841|CH477841](http://www.ncbi.nlm.nih.gov/entrez/query.fcgi?cmd=Retrieve&db=Nucleotide&list_uids=78216666&dopt=GenBank) |
| *Anopheles gambiae α1*a  *Agα1a* | [gb|AAAB01008987.1|](http://www.ncbi.nlm.nih.gov/entrez/query.fcgi?cmd=Retrieve&db=Nucleotide&list_uids=19612317&dopt=GenBank) Anopheles gambiae str. PEST chromosome 2R CRA_x54KRFTDC6T  XM_309723 (mRNA) |
| *Anopheles gambiae α1*b  *Agα1b* | [gb|AAAB01008823.1|](http://www.ncbi.nlm.nih.gov/entrez/query.fcgi?cmd=Retrieve&db=Nucleotide&list_uids=19611779&dopt=GenBank) Anopheles gambiae str. PEST chromosome 3L CRA_x9P1GAV59CY  XM_309723 (mRNA) |
| *Anopheles gambiae α2*  *Agα4a* | [gb|AAAB01008807.1|](http://www.ncbi.nlm.nih.gov/entrez/query.fcgi?cmd=Retrieve&db=Nucleotide&list_uids=19611723&dopt=GenBank) Anopheles gambiae str. PEST chromosome 2L CRA_x9P1GAV591D XM_308639 (mRNA) |
| *Anopheles gambiae α4*  *Agα4b* | [gb|AAAB01008859.1|](http://www.ncbi.nlm.nih.gov/entrez/query.fcgi?cmd=Retrieve&db=Nucleotide&list_uids=19611897&dopt=GenBank) Anopheles gambiae str. PEST chromosome 2R CRA_x9P1GAV5AFD XM_312152 (mRNA) |
| *Apis mellifera α1*a  *Amα1a* | [gi|71799686|gb|CH472729|CH472729](http://www.ncbi.nlm.nih.gov/entrez/query.fcgi?cmd=Retrieve&db=Nucleotide&list_uids=71799686&dopt=GenBank)  Xm 623217 (mRNA); GENE ID: 408388 LOC408388 |
| *Apis mellifera α1*b  *Amα1b* | [ref|NC_007081.1|NC_007081](http://www.ncbi.nlm.nih.gov/entrez/query.fcgi?cmd=Retrieve&db=Nucleotide&list_uids=66835636&dopt=GenBank)  Xm 391936 (mRNA); GENE ID: 550827 LOC550827 |
| *Apis mellifera α2*  *Amα2* | [ref|NC_007073.1|NC_007073](http://www.ncbi.nlm.nih.gov/entrez/query.fcgi?cmd=Retrieve&db=Nucleotide&list_uids=66830420&dopt=GenBank)  Xm 394991 (mRNA) |
| *Apis mellifera α4*  *Amα4* | [ref|NC_007084.1|NC_007084](http://www.ncbi.nlm.nih.gov/entrez/query.fcgi?cmd=Retrieve&db=Nucleotide&list_uids=66837141&dopt=GenBank)  Xm 394092 (mRNA) |
| *Bombyx mori α1*  *Bmα1* | [gi|56198423|gb|CH380422|CH380422](http://www.ncbi.nlm.nih.gov/entrez/query.fcgi?cmd=Retrieve&db=Nucleotide&list_uids=56198423&dopt=GenBank)  [AB072304](http://www.ncbi.nlm.nih.gov/entrez/viewer.fcgi?db=nucleotide&val=19773419) (mRNA) |
| *Bombyx mori α2*  *Bmα2* | [gi|56197945|gb|CH380900|CH380900](http://www.ncbi.nlm.nih.gov/entrez/query.fcgi?cmd=Retrieve&db=Nucleotide&list_uids=56197945&dopt=GenBank)  [AB072305](http://www.ncbi.nlm.nih.gov/entrez/viewer.fcgi?db=nucleotide&val=19773421) (mRNA) |
| *Bombyx mori α3*  *Bmα3* | [gb|AADK01000142.1|](http://www.ncbi.nlm.nih.gov/entrez/query.fcgi?cmd=Retrieve&db=Nucleotide&list_uids=54109570&dopt=GenBank)  [AB072306](http://www.ncbi.nlm.nih.gov/entrez/viewer.fcgi?db=nucleotide&val=19773423) (mRNA) |
| *Drosophila melanogaster* 84B *Dmα1a* | [gnl|dmel|3R](http://flybase.bio.indiana.edu/blast/getSequence.html?jobHash=1ff1c2e93876fd9bd8020cae9bc94f0d&seqId=gnl|dmel|3R) type=chromosome; loc=3R:1..27905053; ID=3R; release=r4.3 NP_476772 (mRNA) |
| *Drosophila melanogaster* 85E *Dmα2* | [gnl|dmel5|3R](http://flybase.bio.indiana.edu/blast/getSequence.html?jobHash=dcb4070a4e6bf681c2b12b06f02d3a3e&seqId=gnl|dmel5|3R) assembled 2005-1-18 md5sum=6b279651b3b268f11e0dd1d87ded0ccc  NM_079573 (mRNA) |
| *Drosophila melanogaster* 84D *Dmα1b* | [gnl|dmel|3R](http://flybase.bio.indiana.edu/blast/getSequence.html?jobHash=8fbf7d5328e9742c39e7d5a103623381&seqId=gnl|dmel|3R) type=chromosome; loc=3R:1..27905053; ID=3R; release=r4.3  NM_079540 (mRNA) |
| *Drosophila melanogaster* 67C *Dmα4* | [gnl|dmel|3L](http://flybase.bio.indiana.edu/blast/getSequence.html?jobHash=9e6265748e440a354e2d9ed6b7c10194&seqId=gnl|dmel|3L) type=chromosome; loc=3L:1..23771897; ID=3L; release=r4.3;  NP_524009 (mRNA) |
| *Drosophila ananassae* 84B  *Daα1* | [gi|91850121|gb|AAPP01019513|AAPP01019513](http://www.ncbi.nlm.nih.gov/entrez/query.fcgi?cmd=Retrieve&db=Nucleotide&list_uids=91850121&dopt=GenBank) |
| *Drosophila ananassae* 85E  *Daα2* | [gi|91854163|gb|AAPP01018383|AAPP01018383](http://www.ncbi.nlm.nih.gov/entrez/query.fcgi?cmd=Retrieve&db=Nucleotide&list_uids=91854163&dopt=GenBank) |
| *Drosophila ananassae* 67C  *Daα4* | [gi|91851249|gb|AAPP01019275|AAPP01019275](http://www.ncbi.nlm.nih.gov/entrez/query.fcgi?cmd=Retrieve&db=Nucleotide&list_uids=91851249&dopt=GenBank) |
| *Drosophila erecta* 84B  *Deα1a* | [gi|91890289|gb|AAPQ01006440|AAPQ01006440](http://www.ncbi.nlm.nih.gov/entrez/query.fcgi?cmd=Retrieve&db=Nucleotide&list_uids=91890289&dopt=GenBank) |
| *Drosophila erecta* 85E  *Deα2* | [gi|91890260|gb|AAPQ01006469|AAPQ01006469](http://www.ncbi.nlm.nih.gov/entrez/query.fcgi?cmd=Retrieve&db=Nucleotide&list_uids=91890260&dopt=GenBank) |
| *Drosophila erecta* 84D  *Deα1b* | [gi|91890289|gb|AAPQ01006440|AAPQ01006440](http://www.ncbi.nlm.nih.gov/entrez/query.fcgi?cmd=Retrieve&db=Nucleotide&list_uids=91890289&dopt=GenBank) |
| *Drosophila erecta* 67C  *Deα4* | [gi|91890166|gb|AAPQ01006563|AAPQ01006563](http://www.ncbi.nlm.nih.gov/entrez/query.fcgi?cmd=Retrieve&db=Nucleotide&list_uids=91890166&dopt=GenBank) |
| *Drosophila grimshawi* 84B  *Dgα1* | [gi|91901577|gb|AAPT01019321|AAPT01019321](http://www.ncbi.nlm.nih.gov/entrez/query.fcgi?cmd=Retrieve&db=Nucleotide&list_uids=91901577&dopt=GenBank) |
| *Drosophila grimshawi* 85E  *Dgα2* | [gi|91900067|gb|AAPT01020831|AAPT01020831](http://www.ncbi.nlm.nih.gov/entrez/query.fcgi?cmd=Retrieve&db=Nucleotide&list_uids=91900067&dopt=GenBank) |
| *Drosophila grimshawi* 67C  *Dgα4* | [gi|91900324|gb|AAPT01020574|AAPT01020574](http://www.ncbi.nlm.nih.gov/entrez/query.fcgi?cmd=Retrieve&db=Nucleotide&list_uids=91900324&dopt=GenBank) |
| *Drosophila mojavensis* 84B  *Doα1* | [gi|91921744|gb|AAPU01011039|AAPU01011039](http://www.ncbi.nlm.nih.gov/entrez/query.fcgi?cmd=Retrieve&db=Nucleotide&list_uids=91921744&dopt=GenBank) |
| *Drosophila mojavensis* 85E  *Doα2* | [gi|91921812|gb|AAPU01010971|AAPU01010971](http://www.ncbi.nlm.nih.gov/entrez/query.fcgi?cmd=Retrieve&db=Nucleotide&list_uids=91921812&dopt=GenBank) |
| *Drosophila mojavensis* 67C  *Doα4* | [gi|91921220|gb|AAPU01011563|AAPU01011563](http://www.ncbi.nlm.nih.gov/entrez/query.fcgi?cmd=Retrieve&db=Nucleotide&list_uids=91921220&dopt=GenBank) |
| *Drosophila persimilis* 84B  *Dpα1* | [gi|80982437|gb|CH479185|CH479185](http://www.ncbi.nlm.nih.gov/entrez/query.fcgi?cmd=Retrieve&db=Nucleotide&list_uids=80982437&dopt=GenBank) |
| *Drosophila persimilis* 67C  *Dpα4* | [gi|80982403|gb|CH479219|CH479219](http://www.ncbi.nlm.nih.gov/entrez/query.fcgi?cmd=Retrieve&db=Nucleotide&list_uids=80982403&dopt=GenBank) |
| *Drosophila pseudoobscura* 84B  *Duα1* | [gnl|dpse|2](http://flybase.bio.indiana.edu/blast/getSequence.html?jobHash=af480302356f4e4d469e5820bff8fe32&seqId=gnl|dpse|2) type=chromosome; loc=2:1..30794189; ID=2; release=r2.0 |
| *Drosophila pseudoobscura* 67C  *Duα4* | [gnl|dpse|XR_group8](http://flybase.bio.indiana.edu/blast/getSequence.html?jobHash=f78f19049ec34fd18028485d3988239b&seqId=gnl|dpse|XR_group8) type=chromosome; loc=XR_group8:1..9212921; ID=XR_group8; release=r2.0 |
| *Drosophila sechellia* 84B  *Dcα1a* | [gi|80980794|gb|CH480821|CH480821](http://www.ncbi.nlm.nih.gov/entrez/query.fcgi?cmd=Retrieve&db=Nucleotide&list_uids=80980794&dopt=GenBank) |
| *Drosophila sechellia* 85E  *Dcα2* | [gi|80980800|gb|CH480815|CH480815](http://www.ncbi.nlm.nih.gov/entrez/query.fcgi?cmd=Retrieve&db=Nucleotide&list_uids=80980800&dopt=GenBank) |
| *Drosophila sechellia* 84D  *Dcα1b* | [gi|80980794|gb|CH480821|CH480821](http://www.ncbi.nlm.nih.gov/entrez/query.fcgi?cmd=Retrieve&db=Nucleotide&list_uids=80980794&dopt=GenBank) |
| *Drosophila sechellia* 67C  *Dcα4* | [gi|80980800|gb|CH480815|CH480815](http://www.ncbi.nlm.nih.gov/entrez/query.fcgi?cmd=Retrieve&db=Nucleotide&list_uids=80980800&dopt=GenBank) |
| *Drosophila simulans* 84B  *Dsα1a* | [gi|62998561|gb|CM000166|CM000166](http://www.ncbi.nlm.nih.gov/entrez/query.fcgi?cmd=Retrieve&db=Nucleotide&list_uids=62998561&dopt=GenBank) |
| *Drosophila simulans* 85E  *Dsα2* | [gi|62998561|gb|CM000166|CM000166](http://www.ncbi.nlm.nih.gov/entrez/query.fcgi?cmd=Retrieve&db=Nucleotide&list_uids=62998561&dopt=GenBank) |
| *Drosophila simulans* 84D  *Dsα1b* | [gi|62998561|gb|CM000166|CM000166](http://www.ncbi.nlm.nih.gov/entrez/query.fcgi?cmd=Retrieve&db=Nucleotide&list_uids=62998561&dopt=GenBank) |
| *Drosophila simulans* 67C  *Dsα4* | [gi|62998562|gb|CM000165|CM000165](http://www.ncbi.nlm.nih.gov/entrez/query.fcgi?cmd=Retrieve&db=Nucleotide&list_uids=62998562&dopt=GenBank) |
| *Drosophila virilis* 84B  *Dvα1* | [gi|91808697|gb|AANI01017216|AANI01017216](http://www.ncbi.nlm.nih.gov/entrez/query.fcgi?cmd=Retrieve&db=Nucleotide&list_uids=91808697&dopt=GenBank) |
| *Drosophila virilis* 85E  *Dvα2* | [gi|91809894|gb|AANI01016019|AANI01016019](http://www.ncbi.nlm.nih.gov/entrez/query.fcgi?cmd=Retrieve&db=Nucleotide&list_uids=91809894&dopt=GenBank) |
| *Drosophila virilis* 67C  *Dvα4* | [gi|91808535|gb|AANI01017378|AANI01017378](http://www.ncbi.nlm.nih.gov/entrez/query.fcgi?cmd=Retrieve&db=Nucleotide&list_uids=91808535&dopt=GenBank) |
| *Drosophila willistoni* 84B  *Dwα1* | [gi|93541941|gb|CH714126|CH714126](http://www.ncbi.nlm.nih.gov/entrez/query.fcgi?cmd=Retrieve&db=Nucleotide&list_uids=93541941&dopt=GenBank) |
| *Drosophila willistoni* 85E  *Dwα2* | [gi|93541941|gb|CH714126|CH714126](http://www.ncbi.nlm.nih.gov/entrez/query.fcgi?cmd=Retrieve&db=Nucleotide&list_uids=93541941&dopt=GenBank) |
| *Drosophila willistoni* 67C  *Dwα4* | [gi|93543025|gb|CH713694|CH713694](http://www.ncbi.nlm.nih.gov/entrez/query.fcgi?cmd=Retrieve&db=Nucleotide&list_uids=93543025&dopt=GenBank) |
| *Drosophila yakuba* 84B  *Dyα1a* | [gi|84679439|gb|AAEU02002618|AAEU02002618](http://www.ncbi.nlm.nih.gov/entrez/query.fcgi?cmd=Retrieve&db=Nucleotide&list_uids=84679439&dopt=GenBank) |
| *Drosophila yakuba* 85E  *Dyα2* | [gi|84682049|gb|AAEU02000008|AAEU02000008](http://www.ncbi.nlm.nih.gov/entrez/query.fcgi?cmd=Retrieve&db=Nucleotide&list_uids=84682049&dopt=GenBank) |
| *Drosophila yakuba* 84D  *Dyα1b* | [gi|84679665|gb|AAEU02002392|AAEU02002392](http://www.ncbi.nlm.nih.gov/entrez/query.fcgi?cmd=Retrieve&db=Nucleotide&list_uids=84679665&dopt=GenBank) |
| *Drosophila yakuba* 67C  *Dyα4* | [gi|84681926|gb|AAEU02000131|AAEU02000131](http://www.ncbi.nlm.nih.gov/entrez/query.fcgi?cmd=Retrieve&db=Nucleotide&list_uids=84681926&dopt=GenBank) |
| *Nasonia vitripennis α1*  *Nvα1a* | GENE ID: 100116270 LOC100116270 |
| *Nasonia vitripennis α1*  *Nvα1b* | GENE ID: 100123147 LOC100123147 |
| *Nasonia vitripennis α1*  *Nvα1c* | GENE ID: 100119612 LOC100119612 |
| *Pediculus humanus corporis α1*  *Phα1a* | [XM_002429076.1](http://www.ncbi.nlm.nih.gov/nuccore/242017282); GeneID:[8234500](http://www.ncbi.nlm.nih.gov/sites/entrez?db=gene&cmd=Retrieve&dopt=full_report&list_uids=8234500) |
| *Pediculus humanus corporis α1*  *Phα1b* | [XM_002432290.1](http://www.ncbi.nlm.nih.gov/nuccore/242023832) |
| *Pediculus humanus corporis α2*  *Phα2* | [XM_002427183.1](http://www.ncbi.nlm.nih.gov/nuccore/242013040) |
| *Tribolium castaneum* *α1*  *Tcα1* | [gb|AAJJ01000552.1|](http://www.ncbi.nlm.nih.gov/entrez/query.fcgi?cmd=Retrieve&db=Nucleotide&list_uids=73486095&dopt=GenBank)  Xm 961339 (mRNA); GENE ID: 656649 LOC656649 |
| *Tribolium castaneum* *α2*  *Tcα2* | [gb|AAJJ01001136.1|](http://www.ncbi.nlm.nih.gov/entrez/query.fcgi?cmd=Retrieve&db=Nucleotide&list_uids=73485511&dopt=GenBank)  Xm 961314 (mRNA) |
| *Tribolium castaneum* *α3*  *Tcα3* | [gb|AAJJ01002028.1|](http://www.ncbi.nlm.nih.gov/entrez/query.fcgi?cmd=Retrieve&db=Nucleotide&list_uids=73484619&dopt=GenBank)  Xm 961491 (mRNA) |
| *Tribolium castaneum* *α4*  *Tcα4* | [gb|AAJJ01000258.1|](http://www.ncbi.nlm.nih.gov/entrez/query.fcgi?cmd=Retrieve&db=Nucleotide&list_uids=73486389&dopt=GenBank)  Xm 968998 (mRNA) |
