# Supplementary material for: Tubulin evolution in insects: gene duplication and subfunctionalization provide specialized isoforms in a functionally constrained gene family
Source: BMC Evol Biol. 2010 Apr 27;10:113. doi: 10.1186/1471-2148-10-113 (PMC2880298; doi:10.1186/1471-2148-10-113)
Supplement: Additional file 3 — Beta Tubulin Intron Features. Intron representation in insect tubulins, intron phase, length, and splice donor/acceptor sites are presented. The amino acids bracketing the splice site, whether the spice site is within unique 5' and 3' codon sequence, and whether tubulins with the intron share the same sequence is presented, to indicate associations between intron presence/absence and tubulin coding sequence. Key: M = A or C, K = G or T, R = A or G, Y = C or T, W = A or T, S = C or G, V = not T, H = not G. [file 1471-2148-10-113-S3.DOC]

**Additional File 3. Beta Tubulin Intron Features.**

| **ID** | **Found In** | **Phase** | **Length**  **(Mean +/- Sdv)** | **Length**  **(Min, Max)** | **Splice site donor, acceptor** | **Amino acids bracketing**  **Splice Site** | **Unique Coding Sequence**  **Associated with Intron?** | **Do Those with the Intron Share Coding Sequence?** |
| --- | --- | --- | --- | --- | --- | --- | --- | --- |
| A | All but Dipteran B2, PhB2a, PhB2b | 0 | 9774+/-1779*  N=66 | 57  71833 | rr/gt  ag/tt | 19  K/F (all but TcB2b - RF) | No | No |
| B | ApB1, PhB1; ApB2a-d; All B3 | 1 | 654+/-372  N=24 | 51  3640 | ng/gy  wg/nh | 56  G/G (most GG) | No | No |
| C | All B4 | 1 | 905+/-217  N=17 | 58  4335 | vg/gt  ag/sh | 98  G/N (all but NcB2c - SN) | No | No |
| D | PhB2a, PhB2b | 1 | 475+/-560  N=2 | 80  867 | wg/gt  ag/gw | 108  E/G (all but PhB2b - DG) | No | No |
| E | ApB1; ApB2a-d | 2 | 210 +/- 248  N=5 | 71  650 | ag/gt  ag/ga | 121  R/K (all but ApB4, PhB4 (RR), TcB4 (QH)) | No | Yes |
| F | PhB1; DsppB2; NvB2a,b; Dipteran B3; ApB3 | 0 | 98+/-26  N=28 | 60  3644 | ag/gt  ag/gs | 131  Q/G (all) | No | No |
| G | AmB2a | 1 | 70 | 70 | cg/gt  ag/gg | 143  G/A (all GS but AmB2a, ApB2d, NvB2a, PhB2a,b (GA)) | Yes | - |
| H | AeB2 | 1 | 57 | 57 | tg/gt  ag/ga | 145  G/M (all but DsppB4, ApB3 GL, PhB2a,b, TcB2; TcB3b (GF)) | No | - |
| I | ApB2a-d, AmB2a; Dipteran B4; PhB4 | 0 | 2827+/-460  N=22 | 72 7471 | ar/gt  ag/gt | 174 K/V (all) | No | No |
| J | NvB3 | 0 | 85 | 85 | ag/gt  ag/gt | 216 K/V (all KL or KV except PhB3 (RV)) | No | - |
| K | All B4 | 0 | 409+/-22  N=17 | 86  487 | hn/gt  ag/vt | 229 S/L (all SL, SV, SA, SM, SI but NvB2a (CA)) | Yes | No |
| L | NvB1, PhB1; NvB2b; PhB3, | 2 | 362 +/-508  N=4 | 80  1127 | hg/gt  ag/rt | 241 R/F (all) | No | No |
| M | NvB2c | 1 | 263 | 263 | cg/gt  ag/gt | 243 P/G (all) | No | - |
| N | NvB2a, AmB2a | 2 | 69 | 69 | ag/gt  ag/ag | 276 R/G (most RG) | Yes | Yes |
| O | ApB4, PhB4 | 0 | 77  81 | 77  81 | aa/gt  ag/at | 292 Q/M (all) | No | Yes |
| P | PhB1; NvB2c | 2 | 289+/-283  N=2 | 89  489 | hg/gt  ag/rt | 309 R/Y (all but PhB2b (PY)) | No | No |
| Q | ApB3 | 2 | 723 | 723 | ag/gt  ag/gg | 318 IFR/G (all) | No | - |
| R | Dipteran B4 | 1 | 1206+/-117  N=14 | 65  2084 | dg/gt  ag/gh | 319 IFRG/P (all GR except B4s) | No | No |
| S | ApB4, PhB4 | 2 | 95+/-28  N=2 | 75  114 | rg/gt  ag/wt | 339 S/Y (most SY) | Yes | No |
| T | AmB2a, NvB2b | 0 | 85+/-4  N=2 | 83  88 | ag/gt  ag/gw | 375 Q/E (most QE) | No | - |
| U | PhB1 | 2 | 102 | 102 | ag/gt  ag/ac | 390 R/R (all but ApB2d, NvB2c (KR)) | No | - |
| V | NvB2a | 1 | 64 | 64 | ag/gt  ag/gt | 402 G/M (all) | No | - |
| W | TcB2b; ApB3, PhB3; DsppB4, ApB4 | 0 | 130+/-191  N=15 | 65  785 | ar/gt  ag/gw | 426 Q/E (all Q but NvB2c (ET); all but 7 E) | No | No |
