# Supplementary material for: Tubulin evolution in insects: gene duplication and subfunctionalization provide specialized isoforms in a functionally constrained gene family
Source: BMC Evol Biol. 2010 Apr 27;10:113. doi: 10.1186/1471-2148-10-113 (PMC2880298; doi:10.1186/1471-2148-10-113)
Supplement: Additional file 4 — Alpha Tubulin Intron Features. Intron representation in insect tubulins, intron phase, length, and splice donor/acceptor sites are presented. The amino acids bracketing the splice site, whether the spice site is within unique 5' and 3' codon sequence, and whether tubulins with the intron share the same sequence is presented, to indicate associations between intron presence/absence and tubulin coding sequence. Key: M = A or C, K = G or T, R = A or G, Y = C or T, W = A or T, S = C or G, V = not T, H = not G. [file 1471-2148-10-113-S4.DOC]

**Additional File 4. Alpha Tubulin Intron Features.**

| **ID** | **Found In** | **Phase** | **Length**  **(Mean +/- Sdv)** | **Length**  **(Min, Max)** | **Splice site donor, acceptor** | **Amino acids bracketing**  **Splice Site** | **Unique Coding Sequence**  **Associated with Intron?** | **Do Those with the Intron Share Coding Sequence?** |
| --- | --- | --- | --- | --- | --- | --- | --- | --- |
| A | All but DsppA1b, PhA4; AmA4 | 0 | 412+/- 26  N=34 | 46  752 | rr/gt  ag/rt | 1  M/R (all but AmA2) | No | No |
| B | AmA4 | 0 | 85 | 85 | aa/gt  ag/gt | 61 Q/V (most HV or QV) | Yes | - |
| C | BmA1, AmA1a,b, NvA1a-c, ApA1a-c; PhA1; AeA2, BmA2, TcA2, AmA2; allA3; TcA4 | 1 | 715+/-376  N=16 | 45  3043 | hg/gy  ag/ay | 76  D/E (all D, most E) | No | No |
| D | AmA2, PhA2 | 1 | 260+/-237  N=2 | 92  427 | ag/gt  ag/gr | 111 G/K (AgA4 GQ, AmA4 GR, BmA3 GT) | No | No |
| E | BmA2, TcA2; BmA3 | 0 | 787+/-224  N=3 | 341  1039 | ar/gt  ag/gg | 133  Q/G (all but AmA4 (AG)) | No | No |
| F | AmA4 | 2 |  | 74 | ag/gt  ag/at | 139 R/S (all HS but AmA4 (RS)) | Yes | - |
| G | ApA1b,c; AeA2, BmA2, DsppA2, AmA2, PhA2; BmA3, TcA4, | 0 | 307+/-102  N=17 | 65  1437 | rr/gt  ag/rt | 176  Q/V (most QV) | No | No |
| H | AmA2 | 0 | 367 | 367 | aa/gt  ag/gc | 207  E/A (all but AmA2 (QA)) | Yes | - |
| I | AmA4 | 2 | 79 | 79 | aa/gt  ag/ga | 215  S/N (most RN) | Yes | - |
| J | PhA2 | 1 | 69 | 69 | tg/gt  ag/gg | 232  G/Q (most GQ) | No | - |
| K | BmA2, BmA3, TcA4 | 0 | 496+/-235  N=3 | 44  832 | ar/gt  ag/rt | 233  Q/I (all but BmA3, TcA3, AgA4b (AV) and AgA4a (QA)) | No | No |
| L | AmA2 | 1 | 111 | 111 | tt/gt  ag/gc | 269 L/A (all LA, LV except AmA4 (LT)) | Yes | - |
| M | BmA3 | 0 | 281 | 281 | ag/gt  ag/gc | 279 K/A (most KA) | No | - |
| N | BmA2,  AmA4 | 0 | 356+/-258  N=2 | 99  614 | ag/gt  ag/at | 301  Q/M (most QM) | No | Yes |
| O | BmA2, PhA2 | 1 | 111 | 111 | tt/gt  ag/gc | 319 R/G (all G, most RG, HG) | No | Yes |
| P | NvA1b,c; AmA2, PhA2, BmA2, BmA3, TcA4, AmA4 | 0 | 1685+/-1189  N=8 | 76  1039 | ar/gt  ag/gt | 351  K/V (all KI, KV) | No | No |
| Q | NvA1a | 0 | 88 | 88 | ag/gt  ag/gt | 370 K/V (most KV) | No |  |
| R | BmA2 | 2 | 728 | 728 | ag/gt  ag/at | 390 R/L (most RL) | Yes | - |
| S | AeA2 | 0 | 62 | 62 | ga/gt  ag/gc | 403 A/F (all AF) | No | - |
| T | AmA2, PhA2 | 2 | 103 | 103 | tg/gt  ag/gt | 407 W/Y (most WY) | No | No |
| U | BmA3 | 1 | 1606 | 1606 | gg/gt  ag/gc | 410 G/E (all GE except TcA3, AgA4a) | No | - |
| V | AgA4b | 1 | 114 | 114 | ag/gt  ag/gt | 412 G/M (all GM except  AgA4a (GL)) | No | - |

* - except *D. ananassae* (which has the same amino acid sequence as other D.sppA2)
